# Ionization Energies and Redox Potentials of Hydrated Transition Metal Ions: Evaluation of Domain-Based Local Pair Natural Orbital Coupled Cluster Approaches

**DOI:** 10.1021/acs.jctc.1c01267

**Published:** 2022-02-22

**Authors:** Sinjini Bhattacharjee, Miho Isegawa, Miquel Garcia-Ratés, Frank Neese, Dimitrios A. Pantazis

**Affiliations:** † Max-Planck-Institut für Kohlenforschung, Kaiser-Wilhelm-Platz 1, 45470 Mülheim an der Ruhr, Germany; ‡ International Institute for Carbon-Neutral Energy Research (WPI-I2CNER), Kyushu University, 744 Moto-oka, Nishi-ku, Fukuoka 819-0395, Japan

## Abstract

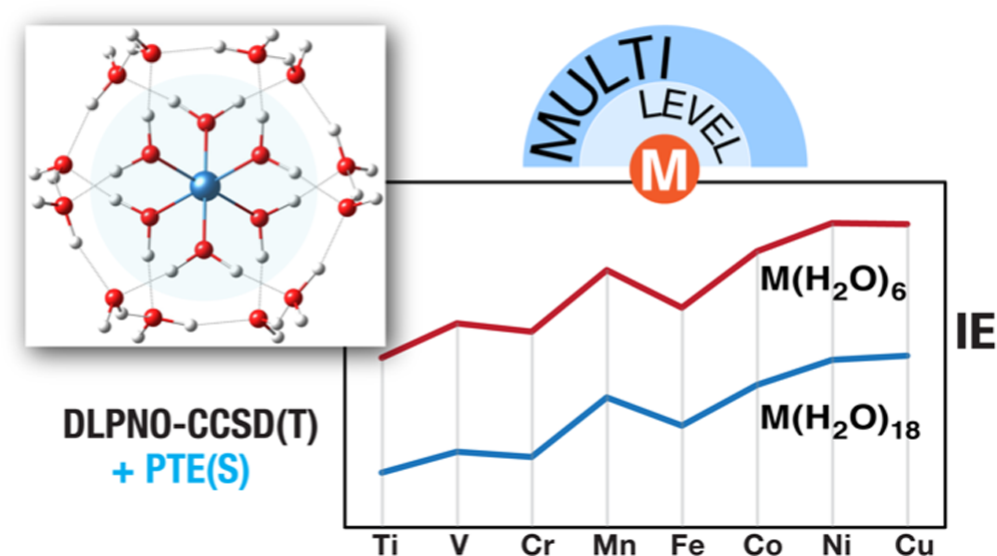

Hydrated transition metal ions are prototypical systems that can be used to model properties of transition metals in complex chemical environments. These seemingly simple systems present challenges for computational chemistry and are thus crucial in evaluations of quantum chemical methods for spin-state and redox energetics. In this work, we explore the applicability of the domain-based pair natural orbital implementation of coupled cluster (DLPNO-CC) theory to the calculation of ionization energies and redox potentials for hydrated ions of all first transition row (3d) metals in the 2+/3+ oxidation states, in connection with various solvation approaches. In terms of model definition, we investigate the construction of a minimally explicitly hydrated quantum cluster with a first and second hydration layer. We report on the convergence with respect to the coupled cluster expansion and the PNO space, as well as on the role of perturbative triple excitations. A recent implementation of the conductor-like polarizable continuum model (CPCM) for the DLPNO-CC approach is employed to determine self-consistent redox potentials at the coupled cluster level. Our results establish conditions for the convergence of DLPNO-CCSD(T) energetics and stress the absolute necessity to explicitly consider the second solvation sphere even when CPCM is used. The achievable accuracy for redox potentials of a practical DLPNO-based approach is, on average, 0.13 V. Furthermore, multilayer approaches that combine a higher-level DLPNO-CCSD(T) description of the first solvation sphere with a lower-level description of the second solvation layer are investigated. The present work establishes optimal and transferable methodological choices for employing DLPNO-based coupled cluster theory, the associated CPCM implementation, and cost-efficient multilayer derivatives of the approach for open-shell transition metal systems in complex environments.

## Introduction

Redox processes involving transition metal ions are important in a wide range of chemical and biological processes. For example, the variation of the redox level on transition metal sites^1,2^ plays an integral role in the function of synthetic catalysts^3−5^ and is at the heart of fundamental enzymatic processes,^6−8^ including the most critical energy converting transformations in biology.^6,9−12^ Obtaining accurate energetics^13−16^ for spin-state and redox changes^17^ in such systems is challenging, and the treatment of the electronic structure problem places heavy demands both on the definition of the computational model in terms of the appropriate representation of the coordination environment^18,19^ and on the electronic structure method.^20−26^ The latter problem is particularly acute in view of the significant errors that can be encountered for larger transition metal systems.^13,21,27^ Ionization energies (IEs)^28−30^ and redox potentials^14,17,20,28−38^ are in this respect crucial target properties that can be used to evaluate the capabilities and limitations of the different components that define the computational approach.

Density functional theory (DFT) methods^21,31,33,36−45^ in conjunction with implicit solvation models^40,46−49^ are widely used for describing transition metal systems owing to simplicity, low cost, and often satisfactory performance of appropriately chosen functionals within sets of closely related chemical systems.^33,50,51^ Nevertheless, DFT has limitations when dealing with complex electronic structure situations such as those encountered in open-shell transition metal complexes.^21,52,53^ Numerous studies have highlighted the role of modern wave-function-based methods to address the challenge of spin-state or redox energetics in transition metal systems,^54−59^ and it is expected that such approaches may become a standard component of a future robust and generally applicable theoretical protocol.^60,61^ Radoń et al. applied multireference calculations (CASPT2^62^ and NEVPT2^63,64^) in studying ligand field transitions of aqua complexes of the first-row transition metal ions,^54,65^ pointing out that benchmark studies on transition metal clusters are prone to significant errors arising from the choice of solvation strategy. Noodleman and co-workers^37^ applied a cluster model to Mn^2+^/Mn^3+^ and Fe^2+^/Fe^3+^ pairs in aqueous solution and showed the importance of including explicit water molecules in the second solvation shell to increase the accuracy of predicted redox potentials. Uudsemaa et al.^36^ also applied the cluster approach and pointed out discrepancies between experimental data and theoretical predictions for the spin state of Co and Ni ions in an aqueous solution. Wang et al. calculated redox potentials with a quantum mechanics/molecular mechanics (QM/MM) approach for 3d transition metals and highlighted the importance of solute–solvent hydrogen bonding.^35^ In addition, previous studies reported the use of wave function theory in calculations of gas-phase ionization energies and aqueous redox potentials using continuum solvation models for organic systems.^32^ Studies of ionization energies and redox potentials have highlighted that the results can be significantly improved if coupled cluster theory^66−68^ is used in place of DFT to calculate changes in electronic energies. In particular, gas-phase ionization energies were shown to improve considerably using coupled cluster theory.^60,66,68,69^

The most popular way to compute solvation energies is through implicit solvation models.^46^ An example is the polarizable continuum model (PCM)^70−72^ and its different variants, such as the conductor-like PCM (CPCM).^73,74^ Within the PCM, the solute–solvent interaction is represented by a collection of charges spread over the surface of a cavity that contains the solute. Although PCM describes electrostatic solvation effects, the nonelectrostatic solvation component of the solvation process can be calculated by means of the solvation model based on density^75^ (SMD). A more complicated scheme is the conductor-like screening model for realistic solvents (COSMO-RS),^76^ which combines quantum mechanics with statistical thermodynamics. The bare CPCM, the SMD, or the COSMO-RS model have been used in combination with DFT to predict aqueous oxidation potentials or interaction energies of organic compounds.^45,47,77^ Studies on organic molecules showed that both COSMO^78^ and SMD^75^ perform similarly for the solvation energy of neutral species but the accuracy is compromised with increasing charge, making the solvation energy the limiting factor in achieving the same level of accuracy for redox potentials as for ionization energies.^32^

For systems with specific solute–solvent intermolecular interactions, the nature of the solvent molecules is clearly different in the first solvation shell than in the bulk of the solvent. This aspect is not properly taken into account by implicit solvation models but can be addressed to some extent by the use of explicitly solvated cluster models, in which a number of solvent molecules that coordinate to the solute are treated at the same quantum chemical footing as the solute. At the same time, the treatment of the solvent as an unstructured continuum with a fixed dielectric constant can introduce severe errors, particularly in cases of specific solute–solvent interactions such as hydrogen bonding in protic solvents.^35^ In principle, one can use an extensive multistep QM/MM approach to deal with the short-range interactions where more layers of solvent can be included;^79^ however, even with minimal inclusion of a single additional layer of solvent molecules, the improvement in the results can be impressive in cases of strong solvent–solute coupling.^34,79−82^ Such is the case for the transition metal cations in an aqueous solution that form the subject of the present work.

Explicitly solvated systems are difficult to model and might even be impractical in combination with expensive electronic structure methods. The recent availability of a near linear-scaling local correlation method for open-shell systems, the domain-based local pair natural approach to coupled cluster theory with singles, doubles, and perturbative triples, DLPNO-CCSD(T),^83−86^ paves the way for more affordable and, at the same time, accurate calculations of redox processes of even larger systems. As demonstrated in the study by Isegawa et al., improved gas-phase ionization energies do not necessarily translate into improved aqueous redox potentials because the continuum solvation model may dominate as the main source of error for calculated redox potentials.^32^ In the demanding case of aqueous transition metal complexes, a major contributing factor is the change in the solvation free energy accompanying the redox process.

The present work focuses on adiabatic ionization energies and redox potentials of explicitly hydrated 3d transition metal ions using the DLPNO-CCSD(T) method that was shown to recover most of the canonical correlation energy at a fraction of the computational cost compared to the canonical coupled cluster approach.^69,87−89^ Since solvation-related errors tend to overshadow the improved electronic energies obtained from a reliable wave function method, we explicitly include a second solvation sphere, which allows a consistent treatment of close-range interactions at the same high level of electronic structure theory rather than by a continuum model. We place emphasis on the dependence of ionization energies on the explicit second solvation shell. We further use the recently implemented perturbation theory energy scheme with singles PTE(S)^90^ as a protocol to compute solvation free energies for the oxidized and reduced species at the coupled cluster level and thus derive standard redox potentials self-consistently. Correlations with experimental redox potentials are discussed. The conditions for obtaining systematically converged results are not self-evident; therefore, we examine several methodological parameters that may affect the accuracy and reliability of the approach. Among others, we investigate the role of different implementations of the perturbative triple excitation corrections,^83,84,91^ and we also analyze the convergence of ionization energies with respect to the dimension of the PNO space.^92,93^ A major challenge involves establishing a suitable approach to deal with even bigger transition metal clusters, such as systems with more extended explicit solvent shells. Toward this goal, we demonstrate a multilayer DLPNO-CCSD(T) approach in which different PNO accuracy thresholds are employed for different regions/shells of a system^94^ and show that this approach holds great promise for the cost-effective treatment of large systems.

## Theory and Methodology

### Explicitly Hydrated Models

**Figure 1:**
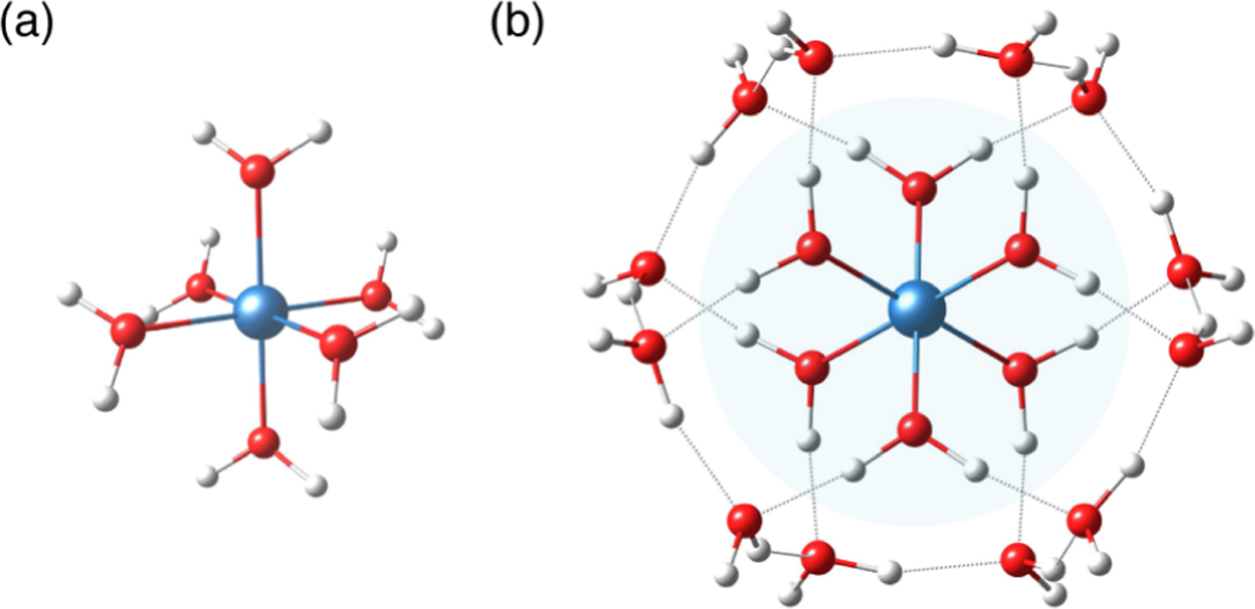
(a) Structure corresponding to the cluster model of [M(H_2_O)_6_]^2+/3+^ in which six water molecules coordinate to the central metal ion (**M-W6**). (b) Explicitly solvated cluster models of [M(H_2_O)_6_·(H_2_O)_12_]^2+/3+^ (**M-W18**) [M = Ti, V, Cr, Mn, Fe, Co, Ni, Cu]. The most stable structure has been considered.

The adiabatic ionization energies (IEs) in aqueous solution for the first-row transition metals Ti–Cu are considered in the current study using (i) 6-water coordinated models (**M-W6**) and (ii) 18-water coordinated models (**M-W18**) (Figure 1). The first type of model involves 6 water molecules directly bonded octahedrally to the metal ion, whereas the latter further incorporates an explicit second shell of 12 additional water molecules. These 12 water molecules are hydrogen-bonded to the first solvation shell leading to a [M(H_2_O)_6_(H_2_O)_12_]^*n*+^ system. More than one configuration of the 18-water cluster has been considered previously.^36^ Here, we adopt the configuration that was reported by Radoń et al. to be the most stable conformation for such complexes in studies of spin-state energetics.^54,65^

The exact coordination number of the metal ions in an aqueous solution is not always obvious. For example, there have been similar conclusions from both theoretical calculations and experimental observations for four, five, and six water molecules coordinated to copper,^82,95−99^ suggesting they can potentially coexist owing to the very small energy differences involved. However, since hexa-coordination is the most common hydration pattern for the majority of the first-row transition metal ions and to ensure consistency in the present approach, throughout this work, we used models where the transition metal ions have six water molecules in their first coordination sphere.

### Electronic Configurations

The set of eight aqueous transition metal complexes was targeted among others because the experimental redox potential values for most of them are known with reasonable accuracy and most of them may undergo one-electron redox reactions in the chosen oxidation states without other associated chemical activity. In principle, both high- (HS) and low-spin (LS) states are possible for [Cr(H_2_O)_6_]^2+^, [Mn(H_2_O)_6_]^2+^, [Mn(H_2_O)_6_]^3+^, [Fe(H_2_O)_6_]^2+^, [Fe(H_2_O)_6_]^3+^, [Co(H_2_O)_6_]^2+^, [Co(H_2_O)_6_]^3+^, and [Ni(H_2_O)_6_]^3+^. However, experiments suggest that aqua complexes of Cr, Mn, and Fe exist in the HS state, whereas the LS state is predominant for Co(III) ions. In this study, we considered high-spin states for all ions except [Co(H_2_O)_6_]^3+^, and both spin states for [Co(H_2_O)_6_]^2+^ and [Ni(H_2_O)_6_]^3+^, although only the most stable one will be treated at all levels. The charge and corresponding spin multiplicities were kept consistent in the 18 water cluster models as well.

### Geometry Optimizations

All geometry optimizations were carried out with a development version of ORCA 5.0.^100−102^ All calculations were performed with the second-order Douglas–Kroll–Hess Hamiltonian (DKH2) to include scalar relativistic effects.^103−105^ The complexes were optimized with DFT using the hybrid TPSSh^106−108^ functional with D3(BJ)^109−111^ dispersion corrections and the DKH-def2-TZVP(-f)^112^ basis set. Tight convergence and optimization criteria (TightSCF, TightOpt) and a fine grid (Grid6, Gridx6) were used. To speed up the calculations, the RIJCOSX^113−115^ approximation was used in conjunction with the SARC/J fitting basis,^116−122^ which is the decontracted version of the def2/J auxiliary basis sets for elements up to Kr.^123^ The optimized coordinates for the **M-W6** and **M-W18** models are listed in the Supporting Information (SI). The effect of the bulk solvent (H_2_O) on the M–O bond lengths was investigated by the conductor-like polarizable continuum model (CPCM). In ORCA, the solvation charges on the surface of the solute cavity are treated as spherical Gaussians through the Gaussian charge scheme together with a switching function to accept or discard them.^124,125^ In particular, we adopt the GVDW scheme. More details on this scheme, that is, the type of solute cavity, number of charges per sphere, and radii for the spheres in the cavity can be found in the paper by Garcia-Ratés et al.^125^ The CPCM scheme adopted in the DLPNO-CCSD(T) calculations is described in the corresponding subsection below.

### Electronic Structure Calculations

For the DLPNO-CC calculations, Kohn–Sham determinants computed with the DFT-TPSSh functional were used as reference. This choice was found by experience to be associated with more well-behaved convergence of the CC calculations. It is noted that we give up on Brillouin’s theorem due to this choice but the emerging off-diagonal Fock matrix elements are properly taken into account by the ORCA implementation. To avoid any misconception, it is stressed that despite the fact that the reference determinant is a DFT determinant, the final DLPNO-CC energy does not contain any DFT component whatsoever. The second-order DKH2 Hamiltonian^103,104^ was employed in all calculations. For open-shell molecules, the energy was obtained on the basis of quasi-restricted orbitals (QROs).^87^ The perturbative triple excitations were computed using the recently published iterative T_1_ algorithm for both closed-shell^126^ and open-shell systems.^83,84,91^ All self-consistent field (SCF) calculations were performed in the absence of any approximations with a convergence criterion of 10^–9^ hartree (VeryTightSCF). The 3s and 3p outer-core orbitals were included in the correlation treatment, while the 1s and 2s inner-core orbitals were kept frozen.^127^ The large automatically generated “AutoAux” fitting basis set^128^ was used where required in correlated wave function calculations. The three truncation parameters *T*_CutPNO_, *T*_CutPairs_, and *T*_CutMKN_, which define cutoffs for the occupation numbers in the pair natural orbitals, for the estimated pair correlation energies, and for the fitting domain selection, were chosen according to built-in settings, using the NormalPNO and TightPNO defaults. For each model (**M-W6**, **M-W18**), the correlation consistent triple ζ basis set cc-pwCVTZ-DK^28,122^ was used on the metal and cc-PVTZ-DK^129−132^ for the rest of the molecule.

For a more detailed quantitative analysis of the DLPNO-CCSD(T) results, we used the open-shell variant of the local energy decomposition (LED) scheme^133−135^ to obtain the inter-fragment energy terms for the individual layers of solvation. This approach quantifies the relative contributions of the metal, the first solvation sphere, and the rest of the cluster, respectively, to the final energy difference for the redox pairs.

Recently, a systematic method to approach the complete PNO space limit in DLPNO-CCSD(T) calculations was proposed.^92^ The correlation energies obtained by varying the *T*_CutPNO_ threshold parameters were extrapolated using a two-point extrapolation scheme, keeping all other parameters of the DLPNO calculations to the default TightPNO settings. The best fit for the dependence of the correlation energy on the parameter *X* (where *T*_CutPNO_ = 10^–*X*^) is of the following functional form
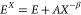
1Here, we tested this approach to investigate the dependence of the DLPNO-CCSD(T) ionization energies on the dimension of the PNO space (*T*_CutPNO_ = 10^–*X*^, where *X* = 5, 6, 7, and 8) using the Fe systems as a test case. The two-point extrapolated energy can be represented as
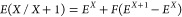
2We use *F* = 1.5 for the current work, as suggested originally.^92^

### Calculation of Ionization Energies and Redox Potentials Using DLPNO and CPCM

Throughout this work, the adiabatic ionization energy (IE) of the transition metal is defined as the difference of the total electronic energy between the **M**^**3+**^ and **M**^**2+**^ form (in eV), computed at the DLPNO-CCSD(T) level of theory, without further thermodynamic corrections.
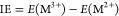
3The aqueous reduction potential (*E*^0^) of the metal ion is defined as
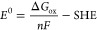
4where
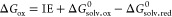
5Reduction potentials are generally tabulated as standard half-cell potentials against a standard reference electrode. Considerable effort has been put toward establishing the absolute electrochemical half-cell standard hydrogen electrode (SHE) potential in different solvents, and different values in the range from 4.24 to 4.73 V have been suggested in the literature.^17,136^ Here, we employ the value of 4.28 V (excluding surface potential), which is the most recommended value.^137^ We obtain the above solvation free energy term (Δ*G*_ox_) directly from DLPNO-CPCM computations. An accurate estimation of the solvation free energies^30^ for the oxidized and reduced species will lead to an accurate prediction of the standard electrode reduction potentials for each redox pair, and the energy obtained is assumed to contain intrinsically the correction to the solvation free energies for the oxidized and reduced species.^138^ There exist different approaches to include the effect of the solvent in coupled cluster calculations, each of them with a different degree of complexity.^139,140^ The simplest of these schemes is the so-called “perturbation theory energy (PTE)” scheme, where the PCM contributions occur through the reference energy and the Fock matrix (solvated orbitals). A further level is the PTE(S) approach, where S stands for singles, which includes an extra solvation term in the correlation energy with respect to the PTE scheme. Neither the PTE scheme nor the PTE(S) scheme involves explicit corrections to the equations to compute the CC excitations (T amplitudes). In the present study, we use the PTE(S) scheme, which has been recently implemented in ORCA 5.0 for open-shell systems,^90,141^ to compute the solvation free energies both for the oxidized and reduced species. It is noted that the various approximate schemes show a high degree of consistency, and hence, the errors arising from the approximation of the solvation terms in the cluster equations must be very small, much smaller than the errors intrinsic in the implicit solvation schemes.

### Multilevel QM/QM Scheme for Truncation Thresholds

The accuracy of DLPNO-CCSD(T) can also be controlled by fine-tuning the *T*_CutPNO_, *T*_CutPairs_, and *T*_CutMKN_ thresholds.^92,93^ When it comes to larger systems, the cost can still become limiting if high-accuracy settings are applied uniformly. In this work, we demonstrate that in the case of **M-W18** systems one can effectively treat different parts of the molecule at different PNO settings instead of treating the entire molecule at a single level of accuracy.^94^ This can be compared to a multilevel QM/QM approach where the different coordination spheres around the central metal ion are treated with different methods but here the method is the same, albeit with different accuracy settings for each layer. In practice, we divided each **M-W18** model into two hypothetical fragments or layers, based on the fact that the inner solvation shell is expected to be more critical in determining the absolute energies of the different oxidation states than the second shell. Therefore, we assigned the metal along with the six directly coordinated water molecules as the first layer and the rest of the water molecules as the second layer (Figure 1b). TightPNO settings (*T*_CutPairs_ = 10^–5^, *T*_CutPNO_ = 10^–7^, *T*_CutMKN_ = 10^–4^) were assigned to the first layer and NormalPNO (*T*_CutPairs_ = 10^–4^, *T*_CutPNO_ = 3.33 × 10^–7^, *T*_CutMKN_ = 10^–3^) to the second layer. The inter-fragment interaction between the two layers was treated using TightPNO settings. An extension of this approach involved more approximate wave function methods for the low-level layer. Here, we further elaborated on the multilevel scheme by treating the pair energies of the second layer at the second-order Møller–Plesset (MP2)^142,143^ and at the Hartree–Fock (HF) level of theory. Global TightPNO settings and default FrozenCore settings for Orca 5 were used throughout these calculations.

## Results and Discussion

### Geometries

All of the geometry optimizations in this work have been carried out without any symmetry constraints and resulted in an approximately octahedral arrangement of the ligands around the central metal ion (Figure 1). The models under investigation could, in principle, possess molecular symmetry as high as *S*_6_. However, the orbital degeneracies in the ground states for several of the aqua complexes lead to Jahn–Teller distortions. This is seen to result in the axial elongation/compression of the metal–oxygen (M–O) bond lengths in high-spin [Cr(H_2_O)_6_]^2+^, [Mn(H_2_O)_6_]^3+^, [Fe(H_2_O)_6_]^2+^, as well as low-spin [Co(H_2_O)_6_]^2+^, [Ni(H_2_O)_6_]^3+^, and [Cu(H_2_O)_6_]^2+^, respectively. The metal–ligand bond distances corresponding to the inner solvation sphere are listed in Table 1. In addition, the tetragonal distortion is strongest for the ^5^E_g_ ground states in high-spin Cr^2+^ and Mn^3+^ (both d^4^) as well as Cu^2+^ (d^9^), arising from the lifting of degeneracy for the single electron in the e_g_ level (M–L σ antibonding).

**Table 1:** Metal–Ligand Bond Distances for the Bare **M-W6** and **M-W6** Complexes with Implicit CPCM (Water) Solvation, and those for the **M-W18** and **M-W18** Clusters with CPCM Solvation, Respectively^a^

TM ion	spin multiplicity (2*S* + 1)	**M-W6**	**M-W6** + CPCM (H_2_O)	**M-W18**	**M-W18** + CPCM (H_2_O)
Ti^2+^	3	2.196	2.173	2.165	2.18
Ti^3+^	2	2.077	2.036	2.047	2.043
V^2+^	4	2.138	2.128	2.127	2.139
V^3+^	3	2.033	1.995	2.005	2.001
Cr^2+^	5	2.073, 2.376	2.048, 2.359	2.051, 2.416	2.053, 2.475
Cr^3+^	4	1.994	1.967	1.975	1.974
Mn^2+^	6	2.192	2.182	2.173	2.19
Mn^3+^	5	1.956, 2.155	1.934, 2.112	1.933, 2.165	1.930, 2.178
Fe^2+^	5	2.113, 2.148	2.108, 2.130	2.112, 2.127	2.127, 2.142
Fe^3+^	6	2.040	1.996	2.016	2.014
Co^2+^	4	2.098	2.090	2.082	2.095
Co^3+^	1	1.918	1.888	1.901	1.9
Ni^2+^	3	2.076	2.076	2.078	2.079
Ni^3+^ (LS)	2	1.880, 2.020	1.876, 2.036	1.886, 2.064	1.881, 2.073
Ni^3+^ (HS)	4	2.000	1.990	1.970	1.972
Cu^2+^	2	2.005, 2.281	2.001, 2.280	1.987, 2.331	1.988, 2.378
Cu^3+^	3	2.008	1.964	1.978	1.971

a The range of values corresponds to the Jahn–Teller distortion observed in ion complexes with a degenerate ground state. All optimizations were performed at the TPSSh-D3BJ/DKH-def2-TZVP(-f) level of theory.

As expected, metal–ligand bond lengths are shorter for the divalent ions compared to the trivalent ones, which arises from a stronger metal–oxygen interaction in higher oxidation states. From the trends in M–O bond distances in Table 1, one can also draw conclusions about the extent of short-range (explicitly using 12-H_2_O) and effective long-range (implicitly using CPCM) solvation effects. The effect of solvation may be expected to be stronger for metal ions that show more variations in bond lengths. For almost all systems, the use of CPCM leads to contraction of the average metal–ligand bond lengths by ca. 0.04 Å with respect to the unsolvated **M-W6**. For the **M-W18** clusters, a further shortening by ca. 0.02 Å is observed. The slight contraction is usually accompanied by elongation in the O–H bond distances of the directly coordinated water ligands, implying that second-sphere solvation effects might result in first-sphere ligands to coordinate more strongly to the metal ion via the O atoms. The addition of explicit water molecules, however, does not systematically change the metal–ligand distances compared to the implicit case and the trend is more metal-dependent. The observation also highlights the importance of explicit solvation models for a few sensitive cases like Cu^2+^, where the change in geometry is not captured well by the implicit model, which could be a consequence of the Jahn–Teller effect.

### Ionization Energies for **M-W6** Models

In the recent work on the spin-state splitting of similar transition metal systems, an elaborate comparison has been made between the results from DLPNO-CCSD and canonical CCSD calculations.^89^ The error associated with the DLPNO approximation, in principle, should yield absolute energy differences between the divalent and trivalent ions within the limits of chemical accuracy. However, non-negligible errors may arise from the treatment of perturbative triples. Until recently, the semicanonical triples (denoted T_0_) had been the usual option as this method can be implemented quite efficiently for closed- and open-shell systems, leading to reasonably accurate relative energies with respect to the canonical results. However, it has been reported that, particularly for certain open-shell systems,^84,86,87^ the T_0_ results may deviate significantly from canonical triples and relative energy differences can be as high as 4 kcal/mol. Such errors may deteriorate the results for bigger systems like those investigated in our case. As an improvement, the iterative triples DLPNO-CCSD(T_1_), recently implemented in ORCA for both closed- and open-shell species,^84,86^ yield in principle more accurate results on the triples correction.^89^ The absolute energies from the SD, semicanonical (T_0_), and iterative triples correction (T_1_) using the DLPNO approach, are provided in the SI. The behavior of the T_1_ was almost consistent for the divalent and trivalent metal complexes studied here.

If we look into the computed ionization energies, the DLPNO-CCSD(T_1_) results have a mean difference of about 0.4 eV from those of the DLPNO-CCSD values (Table 2). Also, the differences in computed IEs between the semicanonical and T_1_ approaches are rather small. On the other hand, the computational time for T_1_ was significantly higher compared to that of the semicanonical approach. In addition, we note that the open-shell DLPNO-CC calculations of the trivalent species were systematically more expensive than the divalent ones for all of the metals, Co being the sole exception. This is due to the better convergence of the closed-shell algorithm.

**Table 2:** Comparison of M^3+/2+^ Ionization Energies (in eV) at the DLPNO-CCSD(T_1_), DLPNO-CCSD(T_0_), and DLPNO-CCSD Levels for the **M-W6** Clusters^a^

redox pair	DLPNO-CCSD(T_1_)	DLPNO-CCSD(T_0_)	DLPNO-CCSD
Ti^2+^/Ti^3+^	14.38	14.40	14.56
V^2+^/V^3+^	15.69	15.71	15.91
Cr^2+^/Cr^3+^	15.35	15.37	15.61
Mn^2+^/Mn^3+^	17.70	17.73	18.03
Fe^2+^/Fe^3+^	16.26	16.26	16.43
Co^2+^/Co^3+^	18.40	18.51	18.24
Ni^2+^/Ni^3+^	19.47	19.53	20.11
Cu^2+^/Cu^3+^	19.40	19.41	19.75

a All values reported here were computed using the cc-pwCVTZ-DK and cc-PVTZ-DK basis sets on the metal and water ligands, respectively. Default TightPNO thresholds were used throughout.

It is noteworthy that the ground-state electronic configuration has a significant role to play for these species, Co and Ni being the only first-row transition metals frequently reported to exist in low-spin states in their aqueous solutions. Further, trivalent Co is the only closed-shell species in our investigation. For Co, the extent of splitting of the d-orbitals for the divalent and trivalent ions, a coexistence of spin states, and/or dimerization in the solution phase or deprotonation of a water ligand might also be plausible explanations to theoretical predictions being different for the experimentally observed configuration. For the most part of our analysis, we consider the spin state that is electronically more stable.

We also investigated the approach to the complete PNO space^92^ limit of DLPNO-CCSD(T) calculations, with respect to a particular basis set (cc-pwCVTZ-DK and cc-PVTZ-DK in our case) using the [Fe(H_2_O)_6_]^2+/3+^ complex as a test case. The results are tabulated in Table S3. Asymptotic behavior is observed for the computed ionization energies (IEs) on tightening the *T*_CutPNO_ threshold by a factor of 10. We further estimated the values for [Fe(H_2_O)_6_]^2+/3+^ using a two-point extrapolation with the functional form described in eq 2. The mean absolute errors (MAE) of the extrapolated *T*_CutPNO_ = 10^–5^/10^–6^ and *T*_CutPNO_ = 10^–6^/10^–7^ energies with respect to *T*_CutPNO_ = 10^–8^ are represented in Figure S1. The accuracy obtained from the *E*(5/6) extrapolation (16.33 eV) is close to that of *T*_CutPNO_ = 10^–7^. In terms of computation time, there is a systematic scaling observed for both models. For the 6-H_2_O complex, the computational times are doubled with tightening of the PNO threshold from *T*_CutPNO_ = 10^–5^ to 10^–6^ and again from 10^–6^ to 10^–7^. For the larger 18-H_2_O model that contains the second layer of water, the same tightening of the thresholds leads to a steeper, approximately 3-fold increase of the run times for a change of *T*_CutPNO_ from 10^–5^ to 10^–6^ but a lower increase (ca. 1.5×) upon further tightening to 10^–7^.

### Effect of the Second Solvation Sphere

In the quest for an optimal protocol that incorporates a better consideration of the solvent without rendering the computational model inaccessible to correlated wave function methods, a modest step toward explicitly solvated cluster models is to treat an additional solvation sphere at the same quantum chemical level as the solute. In the following, we demonstrate the applicability of the DLPNO-CCSD(T) approach to compute the open-shell systems with a second solvation sphere and show that the obtained results are chemically sound and at the same time computationally affordable. The methods investigated here should be contrasted with DFT-based approaches that typically exhibit strong dependence both on the choice of a specific functional and on the nature of the metal under consideration.

**Figure 2:**
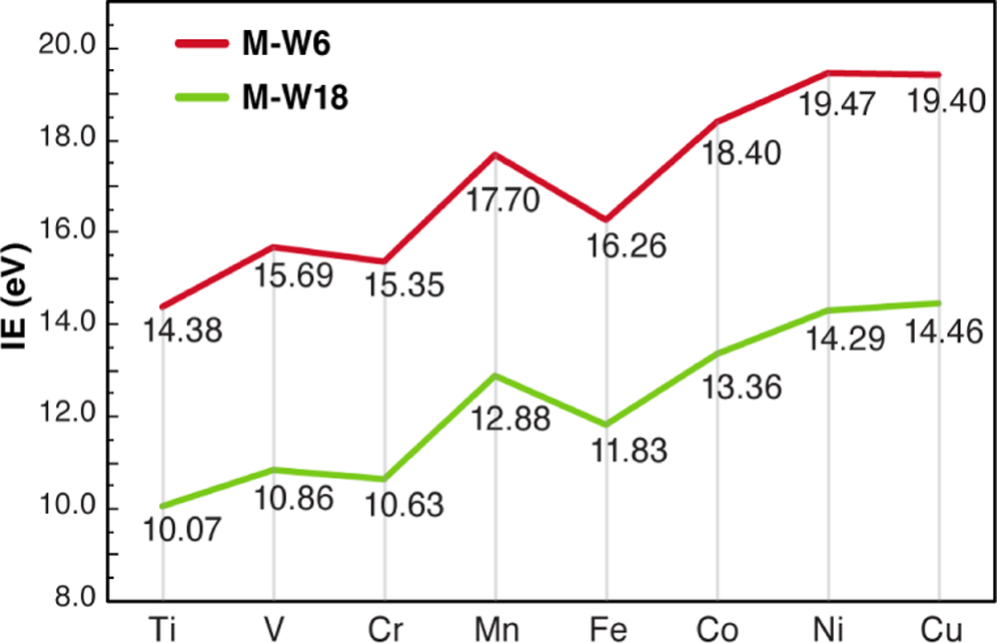
Relative trends in DLPNO-CCSD(T)/TightPNO computed ionization energies (IEs) for each cluster model (**M-W6** and **M-W18**).

The computed ionization energies for each of the metals using DLPNO-CCSD(T_1_)/TightPNO are represented in Figure 2 for both models (**M-W6** and **M-W18**). The IEs show a general increasing trend across the period, as expected from the effective nuclear charges and ground-state electronic configurations of the respective metals. In terms of numerical values, there is a consistent impressive difference of ca. 4–5 eV for the IEs corresponding to the **M-W18** clusters with respect to the **M-W6** values. There can be potentially two effects leading to this observation, namely the change in geometry and/or the electrostatic effects arising from the additional layer of water. However, when the ionization energies were computed using the geometries of the inner M[(H_2_O)_6_] system and excluding the additional layer of water, the effect was negligible. Therefore, we conclude that the significant difference of the IEs on adding the second layer derives predominantly from the electrostatic effect of the second layer that includes hydrogen bonding, an aspect not considered by implicit solvation models.

**Figure 3:**
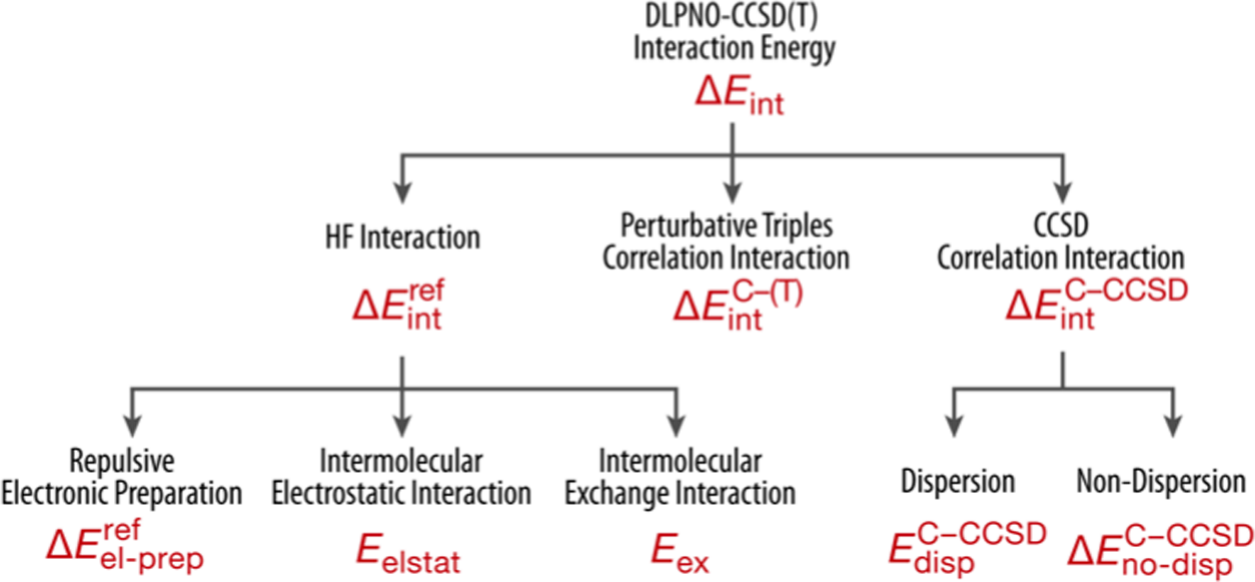
Energy terms in the open-shell DLPNO-CCSD(T)/LED scheme.

To further probe the physical nature of the interaction between the first and second solvation layers, we performed an extensive local energy decomposition (LED) analysis for the specific example of the iron–water clusters. The LED analysis enables a rigorous decomposition of the total interaction energy into contributions arising from the reference (Hartree–Fock) component (Δ*E*_int_^ref^) and the correlation energy, distinguished in the CCSD correlation energy Δ*E*_int_^C-CCSD^, and the perturbative triples correlation energy contribution (Δ*E*_int_^C-(T)^). Figure 3 depicts the distinct components that arise from the decomposition, and are defined as electronic preparation (Δ*E*_el-prep_), electrostatic (*E*_elstat_), and exchange (*E*_ex_) in the case of (Δ*E*_int_^ref^), and dispersive or nondispersive terms for Δ*E*_int_^C-CCSD^. Detailed results for the present test system are provided in the SI (Table S4), while here a summary of salient points will be given.

Not surprisingly, the electrostatic interaction between the individual layers is dominant. Quantitatively, for both systems (with and without the second hydration shell), the electrostatic interactions within the first coordination sphere are stronger for the higher oxidation state by about three times compared to the reduced state (Table S4). It is noted that the combined electrostatic and exchange interactions for the metal (Fe) and the first solvation sphere (six directly coordinated H_2_O molecules) is stronger in [Fe(H_2_O)_6_]^3+^ compared to that in the [Fe(H_2_O)_18_]^3+^ cluster, whereas for the lower oxidation state the interactions are slightly higher in the [Fe(H_2_O)_18_]^2+^ cluster compared to the bare [Fe(H_2_O)_6_]^2+^. Focusing on the 18-water cluster, the decomposition of the total interaction energy (Δ*E*_int_) between the two solvation layers (Table 3) shows that the attractive interactions at the reference level are dominated by the electrostatic terms (*E*_elstat_) for both oxidation states. The electronic preparation term (Δ*E*_el-prep_) however is very high and positive, which basically corresponds to the energy required to distort the electron densities of the individual layers from their ground state. It is important to note that the extent of these contributions depends on the oxidation state of the metal. A similar trend is observed for the correlation energy contributions from CCSD, where the nondispersive terms (Δ*E*_non-disp_), which represent the correction to HF-level electrostatics, are dominant. Hence, the larger stabilization of the Fe^3+^ complex is due to its larger electrostatic interaction compared to Fe^2+^. The contribution from the perturbative triples (Δ*E*^C-(T)^) is comparatively negligible. The decomposition of the final difference in the interaction energy between the two layers of solvation for the Fe^2+/3+^ redox pair is provided in Table 3. We conclude that not only the total but also the individual interaction energy contributions between the coordination layers are dependent on the charge at the metal center. This is in line with the analysis by Wang et al.^35^ that the heterogeneous polarization of the solute electron density and the additional layer of water possibly leads to a decrease in the positive charge at the metal center, thereby lowering the energy difference between the redox pairs.

**Table 3:** Decomposition of Interaction Energies between the Two Layers of Solvation, for the [Fe(H_2_O)_18_] Clusters Using the DLPNO/CCSD(T) LED Scheme^a^

	reference energy			correlation energy		
ion	*E*_elstat_	*E*_ex_	Δ*E*_elprep_	*E*_disp_	Δ*E*_no-disp_	Δ*E*^C-(T)^
Fe^3+^	–12.5691	–1.6003	27.9052	–0.0830	–1.1259	–0.0152
Fe^2+^	–4.6794	–0.6411	19.2412	–0.0558	–0.1666	–0.0126
Δ*E*_int_	–7.8897	–0.9592	8.6639	–0.0272	–0.9593	–0.0026

a All values are in hartree.

### Standard Reduction Potentials Using a Cluster Continuum Approach

**Figure 4:**
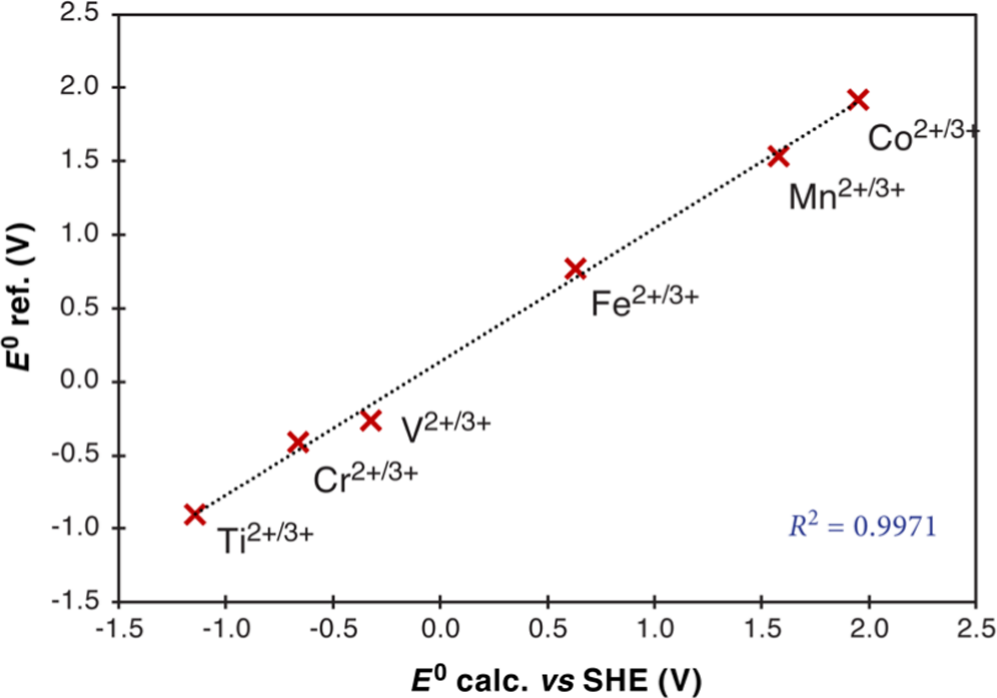
Correlation plot of DLPNO/CCSD(T_1_)/CPCM computed redox potentials with respect to experimental redox potentials for **M-W18** clusters (M = Ti–Co).

The **M-W18** clusters were used to combine DLPNO-CCSD(T) calculations and CPCM with the PTE(S)^90^ scheme in the derivation of solvation energies that were used to estimate the standard reduction potentials of each M^2+^/M^3+^ pair. The free energies of solvation and the calculated redox potentials with respect to the standard hydrogen electrode (SHE) of 4.28 V, for both the **M-W6** and **M-W18** clusters, are provided in Table 4. Figure 4 represents the correlation of the computed values with those reported from the experimental literature.^36,144,145^

**Table 4:** Solvation Free Energies (in eV) and M^3+/2+^ (M = Ti–Cu) Standard Reduction Potentials (in V) Computed Using DLPNO-CCSD(T) in Combination with PTE(S)

	**M-W6**		**M-W18**			
redox pair	Δ*G* solv. (CPCM)	*E*^0^ vs SHE	Δ*G* solv. (CPCM)	*E*^0^ vs SHE	*E*^0^ ref^36,145^	Δ*E*^0^ (W6 – W18)
Ti^2+^/Ti^3+^	–10.40	–0.30	–6.93	–1.14	–0.90	0.84
V^2+^/V^3+^	–10.40	1.01	–6.90	–0.32	–0.26	1.33
Cr^2+^/Cr^3+^	–10.49	0.58	–7.02	–0.66	–0.41	1.24
Mn^2+^/Mn^3+^	–10.49	2.93	–7.02	1.58	1.54	1.35
Fe^2+^/Fe^3+^	–10.32	1.67	–6.92	0.63	0.77	1.04
Co^2+^/Co^3+^	–10.96	3.15	–7.13	1.95	1.92	1.20
Ni^2+^/Ni^3+^ (LS)	–10.81	4.38	–7.05	2.96	a	1.42
Ni^2+^/Ni^3+^ (HS)	–10.65	4.73	–6.98	3.15	a	1.58
Cu^2+^/Cu^3+^	–10.65	4.47	–7.08	3.10	a	1.37

a Experimental values do not exist for these pairs; estimates of 2.3 V for Ni and 2.4 V for Cu have been suggested.^144^

Most remarkably, the absolute solvation free energies are reduced by 3.4–3.8 eV compared to the bare **M-W6** cluster on adding the extra layer of water. This leads to quantitative differences of more than 1 V in the final redox potentials in all cases except Ti between the Δ*E*^0^ computed with the **M-W6** and the **M-W18** models, but there is also an important qualitative distinction in terms of the change of sign for early transition metals. The experimentally observed M^2+^/M^3+^ redox potentials are negative for Ti, V, and Cr, and positive for Mn, Fe, and Co. This is only reproduced here with the **M-W18** models, while for V and Cr the hexa-aqua cluster predicts positive *E*^0^ with respect to SHE. Overall, our estimated values for **M-W6** have a mean absolute error of 1.07 V with respect to reference values, which decrease to 0.13 V on the addition of a second layer of solvation. This result further stresses the importance of clearly identifying sources of error when explicit solvation is not considered to model redox processes in such systems.

It is noted that for Ni and Cu there are no reliably known experimental values but only estimated suggestions (2.3 V for Ni^2+/3+^ and 2.4 V for Cu^2+/3+^). Therefore, we report the computed values here for these two pairs as reference DLPNO-CCSD(T) values without further analysis. Nevertheless, we note that the suggestions do not fit with the computed results, which nicely follow the trends for lighter elements up to Co and are fully consistent with the corresponding IE values.

At this point, it is worth placing these results in the context of past studies that utilized DFT-based cluster continuum methods.^45^ In a well-known study, Noodleman and co-workers reported values of 1.59 and 1.06 V for the Mn^2+^/Mn^3+^ and Fe^2+^/Fe^3+^ couple, respectively.^37^ Our method agrees quite well considering the respective experimental redox potentials reported to be 1.54 and 0.77 V for the two metals. The values reported by Uudsemaa et al. using DFT computations on similar cluster models have a mean difference of 0.3 V from our estimations.^36^ There also have been experimental reports on the spin states of Co^2+^ and Ni^3+^, which stress the low-spin configurations as dominating in aqueous solution. Previous DFT-based studies reported redox potential values for Co^2+^ and Ni^3+^ where the high-spin state shows better agreement with experimental redox potentials. From our calculations, the trend is consistent for Co^2+^. Also, here, one should also keep in mind the comparison of energetics between closed- and open-shell species, which was not always accounted for in previous computational studies. Our results are consistent with the fact that the hydrated Co^3+^ complex should be considered in its low-spin state for better agreement with experimental values. The values obtained with low-spin Ni^3+^ instead give better agreement with previously estimated figures. Furthermore, the energies obtained by the DLPNO-CCSD(T)/CPCM method for low-spin Ni^3+^ complexes are consistently lower than the high-spin counterpart by about 0.2–0.3 eV. As hypothesized, there can be several plausible explanations for this, such as chemical transformations taking place in solution or other sources of error and uncertainty due to the co-existence of dimeric forms or of multiple spin states.

### Evaluation of Multilayer DLPNO-Based Approaches

In the preceding part of our study, we showed that accurate calculations of ionization energies and redox potentials at the full DLPNO-CCSD(T_1_) level can be performed on the complete **M-W18** systems. In this section, we investigate if it is possible to obtain results of equivalent or comparable quality with lower computational cost by introducing approximations in the context of a multilayer approach. In the simplest example, this corresponds to a two-level method for systems consisting of a clearly defined second coordination/solvation sphere, wherein a part of the system assumed to be chemically more important is computed with a higher-accuracy method than the rest of the molecule. The **M-W18** systems are thus divided into an inner fragment (layer 1), consisting of the metal ion surrounded by six water molecules, and an outer layer 2 consisting of the second solvation sphere containing 12 water molecules. Layer 1 is always treated with the DLPNO-CCSD(T_1_) method using TightPNO settings. For layer 2, the following approximations have been considered:

(i) DLPNO-CCSD(T_1_) with NormalPNO thresholds
(ii) Second-order Møller–Plesset (MP2) perturbation theory
(iii) Hartree–Fock (HF) theory

**Figure 5:**
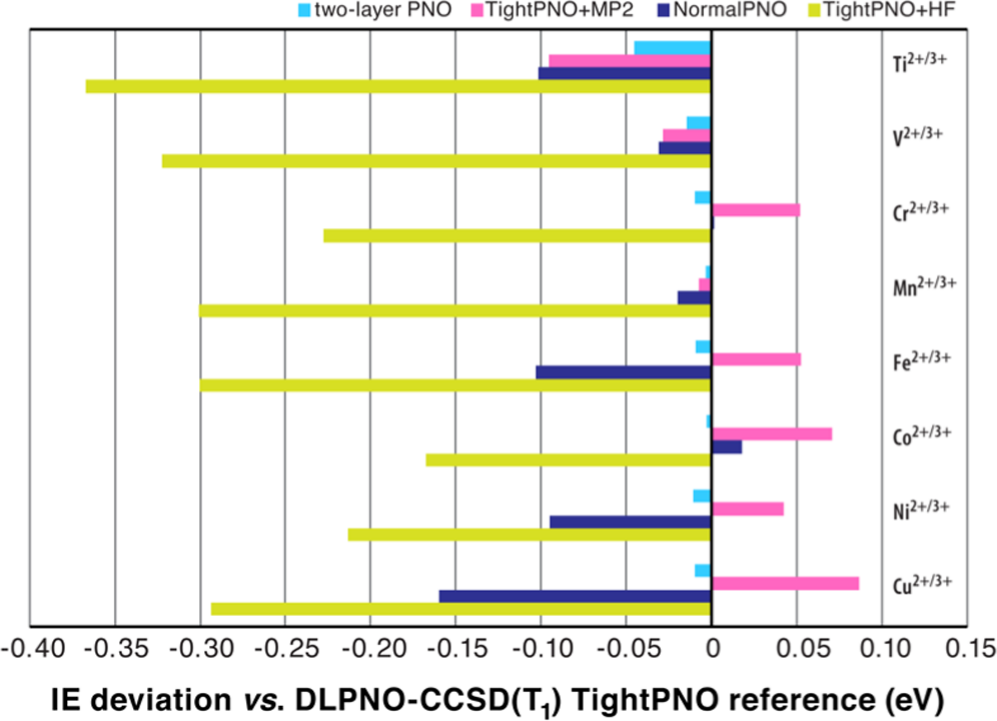
Deviation of M^2+^/M^3+^ ionization energies (in eV) for the **M-W18** clusters computed with the various two-layer approaches discussed in this work and with global NormalPNO DLPNO-CCSD(T_1_) calculations, compared to the reference global TightPNO results.

All of these methods are readily available in ORCA and are accessible through a suitable definition of fragments and the existing multilayer DLPNO machinery (sample input files are provided in the SI). It is important to note that in all of the above two-layer approximations, we chose to treat the more important inter-layer terms needed for the accurate description of the weak interactions with TightPNO thresholds. The resulting IEs are tabulated in Table 5 (absolute energies are provided in the SI) and compared with those obtained from global TightPNO and NormalPNO settings on the entire system. In Figure 5, we compare the errors of the various approaches against the reference global TightPNO result.

**Table 5:** DLPNO-CCCSD(T_1_) Computed Ionization Energies (in eV) for Multilevel Approaches and for NormalPNO, Compared to the Global TightPNO Reference, Ordered by Increasing Mean Signed (MSE) and Mean Absolute (MAE) Errors

redox pair	global TightPNO	two-layer PNO	TightPNO + MP2	global NormalPNO	TightPNO + HF
Ti^2+^/Ti^3+^	10.07	10.02	9.97	9.97	9.70
V^2+^/V^3+^	10.86	10.85	10.83	10.83	10.54
Cr^2+^/Cr^3+^	10.63	10.62	10.69	10.64	10.41
Mn^2+^/Mn^3+^	12.88	12.87	12.87	12.86	12.57
Fe^2+^/Fe^3+^	11.83	11.82	11.88	11.73	11.53
Co^2+^/Co^3+^	13.36	13.35	13.43	13.37	13.19
Ni^2+^/Ni^3+^	14.29	14.28	14.33	14.19	14.07
Cu^2+^/Cu^3+^	14.46	14.45	14.55	14.30	14.17
MSE		–0.013	0.021	–0.061	–0.274
MAE		0.013	0.054	0.066	0.274

The results show that the two-layer PNO approach where the second solvation shell is treated with NormalPNO settings is able to approximate the reference global-TightPNO calculations very well. The two-layer PNO approach has a mean average error of −0.013 eV, with a fairly constant underestimation of the reference IE that is below 0.01 eV, except for the titanium pair. Simultaneously, the fact that the intra-fragment terms of layer 2 are set to the NormalPNO thresholds ensures savings in computational cost compared to a global TightPNO calculation. In the present case, the savings are modest (about 20%) but they would be expected to increase with the increasing size of the second (“low-level”) solvation layer.

The combined DLPNO + MP2 approach is less accurate than the two-layer PNO and shows a larger spread of errors with both positive and negative signs. These errors display a rather regular trend with opposite maxima at the two ends of the series, i.e., underestimation of the IE for the Ti pair by 0.1 eV and overestimation for Cu by almost the same amount. As a result, the method has a mean average error of 0.021 eV but a mean absolute error of 0.054 eV. The cost of this approach for the present system is practically the same as the two-layer PNO approach but it is expected that the cost–benefit will be more prominent with larger systems.

Finally, when the second layer is treated at the HF level the gain in computational cost is more obvious, ca. 1.5 times faster than the global TightPNO reference. However, the errors are considerably higher (−0.27 eV, on average, with a maximum error of −0.37 eV), which highlights the critical importance of treating electron correlation within the second solvation shell (layer 2). The overall deviations in this case are larger than the deviations of the TightPNO DLPNO-CCSD(T) method relative to the reference values. Therefore, the approach cannot be recommended if the goal is to retain the accuracy of the reference method to the largest possible extent.

For comparison, Table 5 includes the results of global NormalPNO DLPNO-CCSD(T_1_) calculations. The global NormalPNO IEs are worse, on average, than those of the two-layer TightPNO + MP2 approach, with a mean average error of −0.061 eV. Nevertheless, the global NormalPNO results remain far superior to those of two-layer TightPNO + HF. Notably, global NormalPNO DLPNO-CCSD(T_1_) calculations are ca. 3 times faster than TightPNO + HF. This suggests that judicious adjustment of PNO cutoffs is the optimal way of balancing both the accuracy and the cost of such calculations. The finer control over errors and convergence afforded by this approach makes it preferable over more conventional “QM/QM” approaches. In view of these results, we expect that multilayer DLPNO-based techniques will find increasing application in the future,^94,146^ not only in the context of explicit solvation but in any computational problem where similar chemically motivated partitions can be made, such as for metalloenzymes and cluster-based simulations of interfacial processes.

## Conclusions

We used the domain-based pair natural orbital implementation of coupled cluster theory to estimate ionization energies and redox potentials of hydrated first-row (3d) transition metal ions in their 2+ and 3+ oxidation states. The systems were modeled with inclusion of an explicit second layer of water molecules, leading to 18 water clusters. Reference values were obtained with the DLPNO-CCSD(T_1_) approach using global TightPNO settings. It was found that the perturbative triple excitations are necessary to obtain accurate ionization energies. The effect of the second hydration shell was quantified in terms of energetics, and the interaction energies were analyzed using the local energy decomposition (LED) scheme for the case of the hydrated iron system. The recent implementation of the conductor-like polarizable continuum model (CPCM) with the PTE(S) scheme was used to determine self-consistent redox potentials at the coupled cluster level. Our results establish conditions for convergence of the DLPNO-CCSD(T_1_) energetics and stress the necessity of explicit consideration of a second solvation sphere, whose effects cannot be simulated by a continuum solvation model. The minimal approach of adding a single layer of water is a major step in the right direction, even if it does not represent a conclusive and definitive treatment of the problem. A more refined computational protocol would have to consider the variability of the solvent shell, the dynamic nature of solvation, and the fact that changes in the coordination number or geometry would necessarily be coupled to the reorganization of the solvation layers. Nevertheless, the present DLPNO-CCSD(T) approach that combines minimal explicit solvation in the form of a second layer of water molecules, plus the PTE(S) model for CPCM, performs robustly and provides reliable estimates of reduction potentials that are within the accuracy of experimental values and largely consistent with previous DFT-based studies. The advantage of the present approach lies in the promise of delivering consistently reliable results for a variety of chemical systems without having to rely on error cancellation, which is a “feature” of DFT-based applications. An important new element of the present study is the multilayer approach to DLPNO-CCSD(T), which was evaluated for three distinct two-layer approaches that retain the high-level treatment of the central core consisting of the metal ion and the directly coordinated water molecules. It was found that an approach that relies on the adjustment of PNO cutoffs for different layers and for their interaction terms represents the most promising way of controlling the accuracy and cost of DLPNO-based calculations on large systems. Thus, the multilayer approach to DLPNO-CCSD(T) paves the way for employing chemically accurate yet computationally affordable local correlation methods in the investigation of more complex open-shell systems, both in the context of explicit solvation and in the case of redox-active molecules and metallocofactors embedded in biological or inorganic matrices.

## Supporting Information Available

Energies (in hartrees) at the DLPNO-CCSD(T1), DLPNO-CCSD(T0), and DLPNO-CCSD levels for the **M-W6** clusters (Table S1); DLPNO-CCSD(T1) ionization energies (in eV) for the **M-W18** and **M-W6** clusters computed with TightPNO and NormalPNO settings (Table S2); ionization energies (in eV) and computation times for DLPNO-CCSD(T1) calculations with TightPNO settings on the Fe clusters, varying the TCutPNO threshold (Table S3); extrapolation to the PNO space limit (Figure S1); decomposition of DLPNO-CCSD(T1) intra-fragment and inter-fragment interaction energies using the open-shell local energy decomposition (LED) approach (Table S4); total energies (in hartrees) from DLPNO-CCSD(T1) on the **M-W18** clusters using different default PNO cutoffs, compared with the two-layer PNO approach (Table S5); total energies (in hartrees) for the **M-W18** clusters using the multilayer approach where the inner layer is computed by DLPNO-CCSD(T1) and the outer layer by MP2 or HF (Table S6); total energies (in hartrees) for the **M-W18** and **M-W6** cluster models using DLPNO-CCSD(T1) (Table S7); and standard redox potentials (V) for the **M-W18** cluster models using TPSSh-D3BJ/DKH-def2-TZVP(-f) with CPCM for water as a solvent (Table S8); sample ORCA input files; and Cartesian coordinates of optimized structures (PDF)
