# Supplementary material for: Ionization Energies and Redox Potentials of Hydrated Transition Metal Ions: Evaluation of Domain-Based Local Pair Natural Orbital Coupled Cluster Approaches
Source: J Chem Theory Comput. 2022 Feb 22;18(3):1619–32. doi: 10.1021/acs.jctc.1c01267 (PMC8908766; doi:10.1021/acs.jctc.1c01267)
Supplement: Supplementary file 1 — ct1c01267_si_001.pdf [file ct1c01267_si_001.pdf]

Supporting Information

for

Ionization Energies and Redox Potentials of Hydrated Transition Metal Ions: Evaluation of Domain-Based Local Pair Natural Orbital Coupled Cluster Approaches

Sinjini Bhattacharjee, Miho Isegawa, Miquel Garcia-Ratés, Frank Neese, Dimitrios A. Pantazis*

Table S1. Energies (in Hartrees) at the DLPNO-CCSD(T_1), DLPNO-CCSD(T_0), and DLPNO-CCSD levels for the **M-W6** clusters. All values were computed using the cc-pwCVTZ-DK and cc-PVTZ-DK basis sets on the metal and the water ligands respectively. Default TightPNO thresholds were used throughout.

Ion	DLPNO-CCSD	DLPNO-CCSD(T_0)	DLPNO-CCSD(T_1)
Ti^{2+}	-1311.338234	-1311.403477	-1311.405776
Ti^{3+}	-1310.802977	-1310.874430	-1310.877354
V^{2+}	-1406.785533	-1406.852080	-1406.854330
V^{3+}	-1406.200746	-1406.274812	-1406.277792
Cr^{2+}	-1508.154268	-1508.220251	-1508.222443
Cr^{3+}	-1507.580543	-1507.655448	-1507.658355
Mn^{2+}	-1615.912777	-1615.977394	-1615.979488
Mn^{3+}	-1615.250218	-1615.325943	-1615.328857
Fe^{2+}	-1729.939295	-1730.006253	-1730.009330
Fe^{3+}	-1729.335346	-1729.408822	-1729.411616
Co^{2+}	-1850.525304	-1850.594256	-1850.598057
Co^{3+}	-1849.821357	-1849.914064	-1849.927872
Ni^{2+}	-1977.808246	-1977.879799	-1977.884867
Ni^{3+}	-1977.069058	-1977.161913	-1977.169433
Cu^{2+}	-2111.904444	-2111.977705	-2111.983668
Cu^{3+}	-2111.178517	-2111.264481	-2111.270592

Table S2. DLPNO-CCSD(T_1) ionization energies (in eV) for the M-W18 and M-W6 clusters computed with TightPNO and NormalPNO settings.

	TightPNO		NormalPNO
**Redox Pair**	**M-W18**	**M-W6**	**M-W18**
Ti^{2+} / Ti^{3+}	10.07	14.38	9.97
V^{2+} / V^{3+}	10.86	15.69	10.83
Cr^{2+} / Cr^{3+}	10.63	15.35	10.64
Mn^{2+} / Mn^{3+}	12.88	17.70	12.86
Fe^{2+} / Fe^{3+}	11.83	16.26	11.73
Co^{2+} / Co^{3+}	13.36	18.40	13.37
Ni^{2+} / Ni^{3+} (LS)	14.29	19.47	14.19
Ni^{2+} / Ni^{3+} (HS)	14.41	19.66	14.28
Cu^{2+} / Cu^{3+}	14.46	19.40	14.30

Table S3. Ionization energies (in eV) and computation times for DLPNO-CCSD(T_1) calculations with TightPNO settings on the Fe clusters, varying the T_{CutPNO} threshold. All calculations were performed on 8 cores with 24GB of memory available per core (%maxcore 24000, in ORCA nomenclature).

	$[Fe(H_2O)_6]^{n+}$			$[Fe(H_2O)_{18}]^{n+}$		
	**Fe(II)**	**Fe(III)**		**Fe(II)**	**Fe(III)**	
T_{CutPNO}	**Time (hh:mm)**	**Time (hh:mm)**	**IE**	**Time (hh:mm)**	**Time (hh:mm)**	**IE**
10^{-5}	01:02	01:18	15.74	06:52	11:43	11.25
10^{-6}	01:44	02:12	16.15	21:35	37:01	11.67
10^{-7}	03:30	04:14	16.26	31:25	55:12	11.83
10^{-8}	06:57	08:06	16.31			

Figure S1. Extrapolation to the PNO space limit. The numbers in the horizontal axis denote the exponent in $T_{CutPNO} = 10^{-x}$.

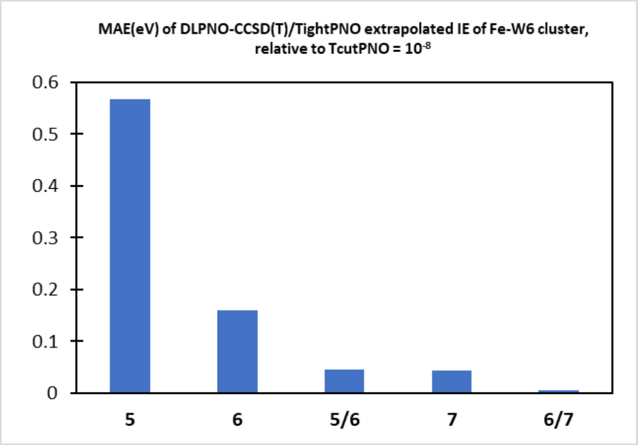

Table S4. Decomposition of DLPNO-CCSD(T_1) intra-fragment and inter-fragment interaction energies using the open-shell local energy decomposition (LED) approach, shown here for the [$Fe(H_2O)_6$] and [$Fe(H_2O)_{18}$] clusters. All values are reported in Hartrees.

	M-W18			M-W6				
	Fe^{3+}	Fe^{2+}	ΔE ($Fe^{3+} - Fe^{2+}$)	Fe^{3+}	Fe^{2+}	ΔE ($Fe^{3+} - Fe^{2+}$)	ΔE $[Fe(H_2O)_{18}]^{3+}$- $[Fe(H_2O)_6]^{3+}$	ΔE $[Fe(H_2O)_{18}]^{2+}$- $[Fe(H_2O)_6]^{2+}$
**Intra-fragment terms**								
**6W**	-447.6343	-453.3414	5.7071	-448.1687	-453.9766	5.8080	0.5343	0.6353
**12W**	-911.3893	-911.9813	0.5920					
**Metal**	-1253.2775	-1256.2922	3.0146	-1253.3711	-1256.3781	3.0070	0.0936	0.0860
**Inter-fragment terms**								
**6W+12W**								
**Electrostatics (REF.)**	-1.1828	-0.8019	-0.3809					
**Exchange (REF.)**	-0.2376	-0.1629	-0.0747					
**Dispersion (strong pairs)**	-0.0396	-0.0346	-0.0050					
**Dispersion (weak pairs)**	-0.0042	-0.0035	-0.0008					
**M+6W**								
**Electrostatics (REF.)**	-10.5449	-3.5861	-6.9589	-10.6421	-3.3617	-7.2804	0.0972	-0.2243
**Exchange (REF.)**	-1.3132	-0.4714	-0.8418	-1.3472	-0.4385	-0.9087	0.0340	-0.0329
**Dispersion (strong pairs)**	-0.0251	-0.0213	-0.0038	-0.0227	-0.0199	-0.0027	-0.0024	-0.0013
**Dispersion (weak pairs)**	-0.0109	-0.0062	-0.0047	-0.0110	-0.0061	-0.0049	0.0001	-0.0002
**M+12W**								
**Electrostatics (REF.)**	-0.8414	-0.2914	-0.5499					
**Exchange (REF.)**	-0.0495	-0.0068	-0.0427					
**Dispersion (strong pairs)**	-0.0005	0.0000	-0.0005					
**Dispersion (weak pairs)**	-0.0027	-0.0015	-0.0012					
**Non dispersion (strong pairs)**	-5.8454	-5.8345	-0.0108	-2.3242	-2.3174	-0.0069	-3.5211	-3.5171
**Non dispersion (weak pairs)**	-0.0107	-0.0201	0.0094	-0.0078	-0.0073	-0.0005	-0.0029	-0.0128

Table S5. Total energies (in Hartrees) from DLPNO-CCSD(T_1) on the M-W18 clusters using different default PNO cutoffs, compared with the two-layer PNO approach.

Ion	TightPNO	NormalPNO	TightPNO (inner layer) + NormalPNO (outer layer)
Ti^{2+}	-2228.564718	-2228.565478	-2228.563701
Ti^{3+}	-2228.194641	-2228.199129	-2228.195298
V^{2+}	-2324.008072	-2324.010292	-2324.006970
V^{3+}	-2323.608894	-2323.612245	-2323.608322
Cr^{2+}	-2425.374684	-2425.377324	-2425.373775
Cr^{3+}	-2424.983895	-2424.986487	-2424.983352
Mn^{2+}	-2533.127992	-2533.131467	-2533.126940
Mn^{3+}	-2532.654837	-2532.659040	-2532.653925
Fe^{2+}	-2647.163857	-2647.165166	-2647.162893
Fe^{3+}	-2646.729097	-2646.734192	-2646.728470
Co^{2+}	-2767.756850	-2767.756659	-2767.755973
Co^{3+}	-2767.266013	-2767.265195	-2767.265272
Ni^{2+}	-2895.041104	-2895.039510	-2895.040208
Ni^{3+}	-2894.516040	-2894.517931	-2894.515547
Cu^{2+}	-3029.141742	-3029.139148	-3029.140923
Cu^{3+}	-3028.610171	-3028.613615	-3028.609825

Table S6. Total energies (in Hartrees) for the M-W18 clusters using the multi-layer approach where the inner layer is computed by DLPNO-CCSD(T1) and the outer layer by MP2 or HF.

Ion	DLPNO-CCSD(T_1)+MP2	DLPNO-CCSD(T_1)+HF
Ti^{2+}	-2229.240336	-2224.823384
Ti^{3+}	-2228.873772	-2224.466803
V^{2+}	-2324.673349	-2320.255126
V^{3+}	-2324.275227	-2319.867793
Cr^{2+}	-2426.156425	-2421.738762
Cr^{3+}	-2425.763743	-2421.356332
Mn^{2+}	-2533.907371	-2529.489929
Mn^{3+}	-2533.434484	-2529.027833
Fe^{2+}	-2647.945096	-2643.527586
Fe^{3+}	-2647.508414	-2643.103861
Co^{2+}	-2768.537875	-2764.120750
Co^{3+}	-2768.044457	-2763.636080
Ni^{2+}	-2895.822069	-2891.405122
Ni^{3+}	-2895.295458	-2890.887895
Cu^{2+}	-3029.923507	-3025.506839
Cu^{3+}	-3029.388947	-3024.986225

Table S7. Total energies (in Hartrees) for the M-W18 and M-W6 cluster models using DLPNO-CCSD(T_1) with the PTE(S) approach and DFT TPSSh with CPCM for water as solvent.

Ion	M-W18	M-W6	TPSSh + CPCM
Ti^{2+}	-2228.796322	-1311.703762	-2231.230348
Ti^{3+}	-2228.680800	-1311.557561	-2231.122027
V^{2+}	-2324.242776	-1407.155310	-2326.686546
V^{3+}	-2324.097335	-1406.961016	-2326.556082
Cr^{2+}	-2425.607730	-1508.523447	-2427.579742
Cr^{3+}	-2425.474815	-1508.344855	-2427.461683
Mn^{2+}	-2533.360460	-1616.278550	-2535.93578
Mn^{3+}	-2533.145154	-1616.013454	-2535.748526
Fe^{2+}	-2647.398223	-1730.311205	-2650.000105
Fe^{3+}	-2647.217780	-1730.092626	-2649.820212
Co^{2+}	-2767.990832	-1850.902157	-2770.616977
Co^{3+}	-2767.761975	-1850.629018	-2770.415469
Ni^{2+}	-2895.276261	-1978.192413	-2897.917418
Ni^{3+} (HS)	-2895.003344	-1977.861423	-2895.003344
Ni^{3+} (LS)	-2895.010271	-1977.874168	-2895.010271
Cu^{2+}	-3029.376245	-2112.292728	-3029.376245
Cu^{3+}	-3029.104969	-2111.971142	-3029.104969

Table S8. Standard Redox Potentials (V) for the M-W18 cluster models using TPSSh-D3BJ/DKH-def2-TZVP(-f) with CPCM for water as solvent.

Redox pair	E^0 vs SHE	E^0 ref
Ti^{2+} / Ti^{3+}	-1.33	-0.90
V^{2+} / V^{3+}	-0.73	-0.26
Cr^{2+} / Cr^{3+}	-1.07	-0.41
Mn^{2+} / Mn^{3+}	0.82	1.54
Fe^{2+} / Fe^{3+}	0.62	0.77
Co^{2+} / Co^{3+}	1.20	1.92
Ni^{2+} / Ni^{3+} (LS)	2.16	2.30
Cu^{2+} / Cu^{3+}	2.09	2.40
MAE		0.45

Sample Inputs:

```
# DLPNO-CCSD(T1) energy calculation, gas phase
# Use !LED in the keyword line to invoke the local energy decomposition (LED) scheme.

!TPSSh cc-pVTZ-DK DKH2 DLPNO-CCSD(T1) autoaux UNO TightPNO VeryTightSCF
!DEFGRID2

%maxcore 20000
%pal nprocs 12 end

%mdci
maxiter 100
maxdiis 15
levelshift 0.5
end

%method
frozencore -10
end

%basis
newGTO Cr "cc-pWCVTZ-DK" end
end

*xyzfile 2 5 filename.xyz
```

```
#DLPNO-CCSD(T1) energy calculation using CPCM

!TPSSh cc-pVTZ-DK DKH2 DLPNO-CCSD(T1) autoaux UNO TightPNO VeryTightSCF
!CPCM(Water) DEFGRID2

%cpcm
CPCMccm 1
end

%maxcore 20000
%pal nprocs 12 end

%mdci
maxiter 100
maxdiis 15
levelshift 0.5
end

%method
frozencore -10
end

%basis
newGTO Cr "cc-pWCVTZ-DK" end
end

*xyzfile 2 5 filename.xyz
```

```
# Multilayer DLPNO-CCSD(T) energy calculation gas phase with definition of fragments
# Use the HFFragInter or MP2FragInter keywords (instead of NormalPNOFragInter) in the
# %mdci block to compute interaction energies at the HF or MP2 level, respectively

!TPSSh cc-pVTZ-DK DKH2 DLPNO-CCSD(T1) autoaux UNO  TightPNO VeryTightSCF DEFGRID2

%maxcore 20000
%pal nprocs 12 end

%mdci
maxiter 100
maxdiis 15
levelshift 0.5
TightPNOFragInter {1 2} {1 1}
NormalPNOFragInter {2 2}
# HFFragInter  {2 2}
# MP2FragInter {2 2}
end

%basis
newGTO Cr "cc-pWCVTZ-DK" end
end

*xyz 2 5
  Cr(1)   0.00000005312509     -0.00000758693890     -0.00000217653280
  O(1)   -0.47057888260532     -1.25991248352848      1.54906005073606
  O(1)   -2.05114140682232      1.19935668251960      0.43675657772371
  O(1)    1.06660947439925      1.24623496024114      1.23339101656542
  O(1)   -1.06660634273380     -1.24625142258114     -1.23339508231532
  O(1)    2.05114390169450     -1.19936658706124     -0.43676187688947
  O(1)    0.47058022527073      1.25989757269669     -1.54906300183221
  H(1)   -1.40962375315646     -1.35295945448651      1.83450051465420
  H(1)    0.12647527860741     -1.37414130451592      2.32311140004353
  H(1)   -2.56208837790882      1.66522286866731     -0.24664292435095
  H(1)   -2.26018341683385      1.59415853515837      1.30302380603656
  H(1)    2.03975045484076      1.34007095504554      1.10949148257859
  H(1)    0.83369052695863      1.38519658121916      2.17855157098154
  H(1)   -2.03974722919243     -1.34008559624118     -1.10949452539550
  H(1)   -0.83369114407843     -1.38520035588326     -2.17855843016404
  H(1)    2.26018991517233     -1.59415831796752     -1.30303257998340
  H(1)    2.56208830529928     -1.66523951574027      0.24663498225071
  H(1)    1.40962496405028      1.35295816163716     -1.83449844314290
  H(1)   -0.12647531172738      1.37414091846063     -2.32311097693409
  O(2)   -3.13760262313716     -1.14109329248007      2.11542841461742
  O(2)    1.36931292550503     -1.24699440814625      3.58339767408322
  O(2)   -3.54196139713703      1.24389256565592     -1.96846579112163
  O(2)   -2.45621142497651      1.27759699643303      3.20222019777118
  O(2)    3.72408560336059      1.17024823316351      0.63058420244848
  O(2)    0.26518377182672      1.21822368928316      3.85667169029597
  O(2)   -3.72408047127101     -1.17026459728635     -0.63057521475083
  O(2)   -0.26518408707900     -1.21820685982544     -3.85667625980514
  O(2)    2.45621074650864     -1.27757484893220     -3.20223100178656
  O(2)    3.54195153451288     -1.24391135057024      1.96846565825789
  O(2)    3.13760764915806      1.14110039861140     -2.11541829624909
  O(2)   -1.36931767121713      1.24700832123940     -3.58339013392702
  H(2)   -3.59848896080644     -1.74045170752891      2.71928512684457
  H(2)   -3.07438965264340     -0.26869917041723      2.57768093971506
  H(2)    1.43909036063679     -1.94571868908232      4.24870252681929
  H(2)    2.22982064534132     -1.24704638590095      3.08310769457125
  H(2)   -3.79099313568717      0.38481447349776     -1.55383293408830
  H(2)   -4.36765529711140      1.67705933211975     -2.22489557117508
  H(2)   -1.54290480205198      1.26809882152917      3.57064900655176
  H(2)   -2.98608778724011      1.83325022799995      3.78976811414601
  H(2)    3.65132939348943      1.09384070028024     -0.35135470715006
  H(2)    4.35251788323253      1.88517952047859      0.80356722654077
  H(2)    0.68488045383538      0.33641775517835      4.01018374826763
  H(2)    0.60971250630114      1.81477718637692      4.53607784385194
  H(2)   -4.35251037438899     -1.88519911238016     -0.80355331043585
  H(2)   -3.65132309326350     -1.09385062392980      0.35136299090401
  H(2)   -0.60970867598080     -1.81475542296449     -4.53608888452329
  H(2)   -0.68488239763840     -0.33640074859822     -4.01018266324736
  H(2)    2.98608520622283     -1.83322244142412     -3.78978591209686
  H(2)    1.54290183637695     -1.26807580823189     -3.57065428850920
  H(2)    4.36764194887830     -1.67708279752318      2.22489897331543
  H(2)    3.79099910808792     -0.38483418844682      1.55383495961630
  H(2)    3.07439352129572      0.26871056237169     -2.57767938166772
  H(2)    3.59849412612458      1.74046416667833     -2.71926953250476
  H(2)   -2.22982671668198      1.24704933737080     -3.08310301719446
  H(2)   -1.43909888674224      1.94573555469947     -4.24869147241460
*
```

Optimized Geometries (gas-phase) using TPSSh-D3BJ/DKH-def2-TZVP(-f)

19

Coordinates for $Co[(H_2O)_6]^{2+}$ HS

Co	0.00000404095949	0.00000392737145	0.00000425475026
O	-0.00906904240180	-0.08845678692774	2.09708815243905
H	0.69227761274723	-0.50042908293383	2.62418827773567
H	-0.57303845967122	0.41178394427979	2.70601787509920
O	-1.52090181890853	-1.44044218600546	0.05745685270352
H	-1.75776344566626	-1.95985446171316	0.84056579226793
H	-1.99444034458394	-1.82013139680633	-0.69792592468340
O	1.52167028007180	-1.44455910829185	0.09971630115584
H	1.39737926747854	-2.40520179181422	0.12958639384450
H	2.44866952876131	-1.28026199734325	-0.13053991222245
O	0.00907210803517	0.08845682335079	-2.09708395661394
H	-0.69227587624390	0.50042561359594	-2.62418526607689
H	0.57303625234413	-0.41179258843167	-2.70601132937014
O	1.52090497846148	1.44044910927054	-0.05745826637001
H	1.75775700434060	1.95986014326594	-0.84057092316741
H	1.99444405288799	1.82014587914946	0.69792035220350
O	-1.52167000045855	1.44455453875432	-0.09971690598765
H	-1.39738732663680	2.40519834527114	-0.12958904528965
H	-2.44866881151674	1.28025107595816	0.13053727758204

19

Coordinates for $Co[(H_2O)_6]^{2+}$ LS

Co	0.00000198597283	-0.00000168869806	-0.00000291759585
O	0.10570602977565	0.13003327871258	2.16432259709527
H	0.65531589599151	-0.47633239599632	2.68415734391494
H	-0.52030305828267	0.53162109216001	2.78549317172080
O	-1.50841933515304	-1.31505279409197	0.03083372695845
H	-1.70030510518873	-1.89374964678968	0.78526054727292
H	-1.74621713411819	-1.79060156173741	-0.78130206837920
O	1.40987980822062	-1.41275414305516	0.11116762990531
H	1.30630968304702	-2.33585631621326	-0.16692192909790
H	2.33661075980398	-1.16267393942993	-0.03368183204159
O	-0.10571099719768	-0.13003217769358	-2.16432474250136
H	-0.65532468770995	0.47633213472269	-2.68415715195671
H	0.52029884563483	-0.53161425238310	-2.78549825265886
O	1.50842158646992	1.31505077294427	-0.03083323582620
H	1.70030715765480	1.89375221026655	-0.78525668389502
H	1.74621136648746	1.79059909987352	0.78130534929569
O	-1.40987561691265	1.41275204587822	-0.11116849769839
H	-1.30630124321576	2.33585452530520	0.16691854393016
H	-2.33660594127996	1.16267375622542	0.03368840155754

19

Coordinates for $Co[(H_2O)_6]^{3+}$ LS

Co	-0.00000007703620	0.00000071711586	-0.00000009551675
O	0.11848238394159	0.14070276131800	1.90832856141629
H	0.61769719437198	-0.54442602749250	2.40034785508057
H	-0.61650463180791	0.44976047434032	2.47622255013643
O	-1.43743987221773	-1.26642132391964	0.07515414824734
H	-1.58977084737151	-1.87001495064295	0.83055743884629
H	-1.70071597067005	-1.73161929139470	-0.74635261031745
O	1.34675729065315	-1.35934840024027	0.13056159923705
H	1.21132307074524	-2.28175019099617	-0.16827357603536
H	2.27993134556864	-1.11617506066801	-0.04498110167126
O	-0.11848230427429	-0.14070342881595	-1.90832790987296
H	-0.61769956533620	0.54442336271010	-2.40034775730888
H	0.61650651012611	-0.44975800660032	-2.47622129242011
O	1.43744065920097	1.26642127025047	-0.07515437299748
H	1.58977268709475	1.87001490405405	-0.83055749785475
H	1.70071567611232	1.73161943893501	0.74635254875393
O	-1.34675763261568	1.35934893961622	-0.13056183984385
H	-1.21132419347423	2.28175060040218	0.16827411280567
H	-2.27993172301097	1.11617421202830	0.04497923931528

19

Coordinates for $Cr[(H_2O)_6]^{2+}$ HS

Cr	-0.00000660420513	-0.0000289940821	-0.00000591952562

O -0.00000635836411 2.37605621111605 -0.00000298603779
H 0.77037722775233 2.96460099908822 0.00001781185342
H -0.77038941186300 2.96460167270548 0.00003647178759
O 0.00000350979300 -0.0000049013233 2.07254790950011
H 0.00001858609778 0.78532900352931 2.64135566187213
H 0.00001790758557 -0.78532902766188 2.64135644755293
O 2.07456154950192 0.00001038675277 -0.00000256512126
H 2.64664656705892 0.00001584835632 0.78213139337328
H 2.64664999089820 0.00001402652258 -0.78213394785768
O -0.00000549566388 -2.37606867958818 0.00000748452543
H -0.77038787878272 -2.96461510320245 0.00002331052724
H 0.77037762240491 -2.96461412943593 -0.00001399652102
O -0.00000282774662 -0.00001162052404 -2.07256012058421
H 0.00001956754085 -0.78534265717568 -2.64136545312543
H 0.00002282006845 0.78531485580138 -2.64137144015368
O -2.07457425478291 0.00000762618964 -0.00000630702847
H -2.64666034618380 0.00001145553200 -0.78213941730812
H -2.64666217110974 0.00002252153494 0.78212566227115

19
Coordinates for Cr[$(H_2O)_6]^{3+}$ HS

Cr 0.00000298647146 -0.00000072382398 0.00000027558049
O -0.00000123072248 1.99440608897255 0.00000055508535
H 0.79083347035687 2.56884909297594 -0.00000637412161
H -0.79083860015963 2.56884601989900 -0.00001069621177
O 0.00000028343732 -0.00000250797968 1.99440710868143
H -0.00001480213051 0.79083322346280 2.56884890467496
H 0.00000496170077 -0.79083915684854 2.56884769044366
O 1.99440920894877 0.00000198210223 0.00000204265978
H 2.56884924307643 -0.00001138842638 0.79083895550682
H 2.56885128274535 0.00000107590212 -0.79083340829069
O 0.00000290712864 -1.99440763514845 0.00000153785789
H -0.79083290573238 -2.56884920017451 -0.00000250793873
H 0.79083900195493 -2.56884918054110 0.00000180136721
O 0.00000253171472 0.00000150687861 -1.99440598248235
H -0.00000322704006 -0.79083423047714 -2.56884796296838
H -0.00001457677780 0.79083861287870 -2.56884629471818
O -1.99440292231800 0.00000095411912 0.00000135101755
H -2.56884411424592 0.00000605392009 -0.79083505594336
H -2.56884349840848 0.00000941230864 0.79083805979991

19
Coordinates for Cu[$(H_2O)_6]^{2+}$

Cu 0.00366746331640 0.00471029549030 -0.00954170392229
O -0.08632105392055 -0.39075032765875 -1.97269475185798
H -0.84970682748467 -0.12803528630764 -2.51199776273952
H 0.70972702247518 -0.35474441386222 -2.52739277113925
O 0.52339506864639 1.91647998110743 -0.28372644652077
H 0.28763213995819 2.43355771054330 -1.07008353734357
H 0.45305459655233 2.50555670452888 0.48546032801729
O 2.24240202023177 -0.43083395869284 -0.03197784778330
H 2.90121895807028 0.27517604580882 -0.11732410958815
H 2.74918626233541 -1.25621743903911 0.00280709329708
O -0.51676549635275 -1.90681914956097 0.26451255434030
H -0.44582188192285 -2.49570974408713 -0.50477085337807
H -0.28068704981420 -2.42383425532592 1.05082449796077
O 0.09358540572223 0.40051432451760 1.95356692671041
H -0.70249845930014 0.36440596964960 2.50820565157233
H 0.85690739109311 0.13760547773705 2.49286782900204
O -2.23494173825632 0.44001534523629 0.01282308699875
H -2.74177564637389 1.26537472689369 -0.02177964283333
H -2.89368654617593 -0.26606737507839 0.09812184390726

19
Coordinates for Cu[$(H_2O)_6]^{3+}$

Cu 0.00383276278505 0.00466294419219 -0.00956985644566
O -0.05620800807352 -0.42272860314397 -1.97210149021669
H -0.86240745765920 -0.15733696994209 -2.46419756400134
H 0.70471867453899 -0.29147702798316 -2.57569520825650
O 0.53648956242387 1.91961287718540 -0.29475145572236
H 0.28156232835591 2.43678620458140 -1.08733332816533
H 0.46957570195645 2.52382821782620 0.47536230054640

O	1.96929191974809	-0.36427101468953	0.16027480090871
H	2.58594952481504	0.34304269876614	-0.12610975975052
H	2.37895065310722	-1.21967776304839	-0.08675084246168
O	-0.52862876006997	-1.91034001982597	0.27511067639981
H	-0.46381560764446	-2.51425272945426	-0.49543159972517
H	-0.27429109501663	-2.42802723632545	1.06756246823622
O	0.06336951607264	0.43238232720465	1.95281567214464
H	-0.69763110980493	0.30084580766181	2.55626683043925
H	0.86973708408789	0.16775515057941	2.44506618138833
O	-1.96157389700784	0.37389263839484	-0.17895099924877
H	-2.37096344026422	1.22934941711440	0.06832073253580
H	-2.57816352335035	-0.33318560309362	0.10813416639488

19

Coordinates for $Fe[(H_2O)_6]^{2+}$ HS

Fe	0.00000107871209	0.00000705577111	0.00001300956672
O	0.00000258038789	0.00000615245991	2.14763922480153
H	0.62472674774217	-0.46393698861910	2.72518498358199
H	-0.62472779302988	0.46393836543157	2.72518698793781
O	-0.79872477620098	-1.95662671413731	0.00000046849958
H	-1.06094969676239	-2.46880244058686	0.78035812333166
H	-1.06094209526609	-2.46879295374956	-0.78036590025231
O	1.99389726488496	-0.78196791700478	0.00000510747787
H	2.23519198472451	-1.72049220832623	-0.00001118328077
H	2.81994281191633	-0.27527450011962	-0.00002684883394
O	0.00000565692300	0.00000679967252	-2.14761990101923
H	-0.62472743473318	0.46393347905080	-2.72516936245085
H	0.62472701669570	-0.46393933912185	-2.72516640568854
O	0.79872688169283	1.95663629112332	0.00000283442021
H	1.06094658166844	2.46880117275830	-0.78036356705721
H	1.06095745050209	2.46880735749582	0.78036152685118
O	-1.99390287646160	0.78195986750466	0.00000173603872
H	-2.23520962423354	1.72048101134077	-0.00001252639827
H	-2.81994175916237	0.27525550905653	-0.00001830752616

19

Coordinates for $Fe[(H_2O)_6]^{2+}$ LS

Fe	-0.00000261218413	-0.00000393572142	0.00000821917535
O	0.00002318563910	0.00001951602791	1.99575795150990
H	0.71316011934033	-0.33328387491991	2.55963385730297
H	-0.71315256755767	0.33323244311705	2.55963872428269
O	-0.60508168618916	-1.90173924863750	0.00001626581694
H	-1.01236961104845	-2.30423387576971	0.78280898219578
H	-1.01233734921603	-2.30424535792514	-0.78278769058453
O	1.84876102367937	-0.75362849144381	-0.00001186161139
H	2.01571647226951	-1.70815590017221	-0.00001057568133
H	2.69776010402886	-0.28922271719437	0.00001186592425
O	-0.00002181612956	-0.00002675775460	-1.99574729647131
H	-0.71315097070954	0.33328917053346	-2.55962573747915
H	0.71315002581921	-0.33324702725148	-2.55962872095053
O	0.60508795389505	1.90172933725864	-0.00000984658224
H	1.01235362687233	2.30423427470621	-0.78280952984954
H	1.01232304264737	2.30425374547388	0.78279614902898
O	-1.84875703902712	0.75363223743009	0.00004245689967
H	-2.01570133222736	1.70816168673122	-0.00002032698652
H	-2.69776056990212	0.28923477551169	-0.00006288593997

19

Coordinates for $Fe[(H_2O)_6]^{3+}$ HS

Fe	-0.00000342470488	0.00000051769151	-0.00000067326372
O	0.00000159781185	2.04005238772406	-0.00000401102227
H	0.78749651234292	2.62036508921003	0.00002008540737
H	-0.78749033284747	2.62036848465307	0.00001746837218
O	-0.00000389629000	0.00000210206943	2.04005156838242
H	0.00002271942736	0.78749607578680	2.62036551950278
H	0.00000709814992	-0.78749059903851	2.62036684003382
O	2.04004848513794	-0.00000498198836	-0.00000172678779
H	2.62036321234443	0.00002880004297	0.78749162801966
H	2.62036229965086	0.00000918335605	-0.78749557837114
O	-0.00000980100119	-2.04005106989079	0.00000604870812
H	-0.78750490112060	-2.62036330143379	-0.00000738634542
H	0.78748199747887	-2.62036745318714	-0.00001205441983

O	-0.00000290288941	-0.00000779494677	-2.04005283621915
H	0.00000067621932	-0.78750386347048	-2.62036374935343
H	0.00002442051345	0.78748284952865	-2.62037092106548
O	-2.04005505166172	0.00000667753367	-0.00000295240177
H	-2.62036842523388	-0.00002234798656	-0.78749704704516
H	-2.62037028332776	-0.00000075565383	0.78748977786882

19

Coordinates for $Mn[(H_2O)_6]^{2+}$ HS

Mn	0.00000201455365	-0.00000048645268	0.00000156633664
O	-0.00000057126177	2.19163825946072	-0.00000008044231
H	0.77758567844488	2.77048572646976	-0.00000325555131
H	-0.77758846595279	2.77048357200541	-0.00000765145389
O	0.00000069520681	0.00000058295339	2.19164079969562
H	-0.00001614195616	0.77758786698746	2.77048697482718
H	0.00000445895833	-0.77758615642643	2.77048765954013
O	2.19164123819105	0.00000111678046	0.00000319728969
H	2.77048721722560	-0.00000496374930	0.77759066365743
H	2.77048839464707	-0.00000894256315	-0.77758339312782
O	0.00000527270215	-2.19163888323955	0.00000058174040
H	-0.77758084656949	-2.77048653380442	-0.00001104695231
H	0.77759333802163	-2.77048395526883	-0.00000646309218
O	0.00000197583027	-0.00000052655129	-2.19163747934883
H	-0.00000785782297	-0.77758757327824	-2.77048392171549
H	-0.00001198888630	0.77758649172515	-2.77048396663186
O	-2.19163712373438	-0.00000350574378	0.00000188085468
H	-2.77048377286941	0.00000699928411	-0.77758510882862
H	-2.77048351472818	0.00001091141120	0.77758904320286

19

Coordinates for $Mn[(H_2O)_6]^{3+}$ HS

Mn	0.00000165795565	0.00000045677071	0.00000070269051
O	0.00000209506528	1.96252945546092	-0.00000108800833
H	0.79494886655348	2.53314918652353	-0.00001874095784
H	-0.79494446422561	2.53314963959791	0.00000474868035
O	0.00000418782228	-0.00000059221186	2.15497783472378
H	-0.00002279706770	0.77760075826340	2.74527847104962
H	-0.00002149211052	-0.77760285786424	2.74527734941866
O	1.95625496610263	-0.00000064286167	-0.00000531712316
H	2.52183414218709	-0.00001615649243	0.79989467916797
H	2.52182860544421	0.00001716905338	-0.79990966349252
O	0.00000217547168	-1.96252916748544	-0.00000019555024
H	-0.79494434816596	-2.53314930248066	0.00002039482273
H	0.79494892285744	-2.53314889684826	-0.00001241973540
O	-0.00000780057271	-0.00000006827731	-2.15497663407700
H	0.00001157635461	-0.77760211845588	-2.74527634618413
H	0.00001065577674	0.77760159430909	-2.74527700811999
O	-1.95625147305384	0.00000035890878	0.00000650556396
H	-2.52183019203528	0.00001668421165	-0.79989398056874
H	-2.52182528435948	-0.00001550012162	0.79991070769979

19

Coordinates for $Ni[(H_2O)_6]^{2+}$

Ni	-0.00001264751237	-0.00000700917223	0.00000675600457
O	0.25501408612814	-0.10226199792173	2.05765406508849
H	0.80723035630582	0.55053693512099	2.51543594766316
H	-0.37696727209250	-0.45235421188400	2.70359015273936
O	1.50581506602294	-1.42383714236284	-0.12618098959801
H	1.82398378075782	-1.87537247819466	0.67101523115136
H	1.69719668226308	-1.99939702670922	-0.88202685416716
O	1.51571820556846	1.41747251274697	0.02416079236583
H	2.42880672331904	1.16076977107200	-0.17876553052522
H	1.42604439823923	2.35932558133719	-0.18615192907833
O	-0.25500580289043	0.10226354520359	-2.05765296138687
H	-0.80721714573878	-0.55052441134805	-2.51545650218252
H	0.37698875053789	0.45236245332271	-2.70357252131949
O	-1.50582459362037	1.42383979054871	0.12617962269815
H	-1.82396943459513	1.87537512495588	-0.67102622849919
H	-1.69720138159295	1.99941280687904	0.88201679741685
O	-1.51573059560911	-1.41748563521552	-0.02416158054987
H	-2.42881895152104	-1.16078428171256	0.17876757660222
H	-1.42605022396975	-2.35933432666629	0.18616815557667

19

Coordinates for Ni[(H$_2$O)$_6$]$^{3+}$ LS

Ni	-0.00000421618040	0.00000287946920	0.00000270921565
O	0.25363455257073	-0.14194412399961	1.85445401890043
H	0.76267714728477	0.59285329080840	2.26661272497407
H	-0.50300227145526	-0.36676686114121	2.43993601045578
O	1.46409976446794	-1.35305954121317	-0.18138342683859
H	1.77131751119836	-1.81524337689535	0.62589830308202
H	1.56096440646144	-1.98572637815331	-0.92172532475987
O	1.45785826914901	1.38759139472076	0.04043693627448
H	2.38404945581056	1.13891512165227	-0.15680487971352
H	1.34497352180077	2.32328667611245	-0.22196569553428
O	-0.25363665182133	0.14194534397558	-1.85445038437882
H	-0.76267557125256	-0.59285421049750	-2.26661012337781
H	0.50300294042928	0.36676770465995	-2.43992900330617
O	-1.46410582263170	1.35306500732583	0.18138221839475
H	-1.77131985932154	1.81524589450762	-0.62590264564938
H	-1.56096651683789	1.98573678544084	0.92172048861161
O	-1.45785489228017	-1.38759543929577	-0.04043575530783
H	-2.38404944018089	-1.13892903761468	0.15680194175385
H	-1.34496232721111	-2.32329112986230	0.22196188720364

19

Coordinates for Ni[(H$_2$O)$_6$]$^{3+}$ HS

Ni	0.00000004298932	-0.00000032675032	-0.00000015556542
O	0.17618338626056	0.00131143637300	1.99268190028720
H	0.85606007921151	0.48182521999829	2.50624655409623
H	-0.42147812003229	-0.44825561524463	2.62308406743516
O	1.36668454766231	-1.45575456135662	-0.09758052099281
H	1.74621756382781	-1.93911658600789	0.66339780866061
H	1.75060456998831	-1.82720004732866	-0.91705666617329
O	1.46490910496394	1.35302940452467	-0.15458596838510
H	2.42490747794146	1.16760169591149	-0.12766325810316
H	1.34282535085794	2.32020332497390	-0.23573973470210
O	-0.17618296051265	-0.00131119660939	-1.99268228797998
H	-0.85606017915610	-0.48182371385819	-2.50624748264215
H	0.42147869813589	0.44825629722397	-2.62308402441979
O	-1.36668445907457	1.45575390881243	0.09757931545245
H	-1.74621761557912	1.93911517917488	-0.66339946453729
H	-1.75060415271988	1.82720049667846	0.91705512722727
O	-1.46490947575492	-1.35302967375553	0.15458793268563
H	-2.42490780170974	-1.16760158193530	0.12766536950139
H	-1.34282605729978	-2.32020366082457	0.23574148815516

19

Coordinates for Ti[(H$_2$O)$_6$]$^{2+}$ HS

Ti	-0.00001532098822	-0.00000566129185	0.00000586066855
O	1.43490621061543	-1.02133318090893	1.30937311948780
O	-0.00004764109720	1.81887765486851	1.23247324202210
O	1.56280784652701	0.76100894129206	-1.34129236152998
O	-1.56282724800304	-0.76101762897978	1.34131620849934
O	0.00002184609707	-1.81889245021050	-1.23245620447525
O	-1.43493744954503	1.02132089541045	-1.30935743611325
H	1.72653137394042	-0.73851033463334	2.18928243846582
H	1.98123712800018	-1.78694622326527	1.07446912614859
H	0.43601287132519	2.66196064169958	1.03688072669486
H	-0.35621549563981	1.89833908784116	2.13058177059393
H	1.47270058494761	1.50363926961532	-1.95791973058133
H	2.49700603172224	0.50363633973400	-1.36016250433607
H	-1.47271270162956	-1.50364530563630	1.95794568838061
H	-2.49702734141530	-0.50365221934273	1.36018767935658
H	-0.43604022320962	-2.66197347265033	-1.03685840294574
H	0.35618680841149	-1.89835929773939	-2.13056541290885
H	-1.72655621437338	0.73850338341611	-2.18927061699837
H	-1.98127251468550	1.78693025978122	-1.07445127442935

19

Coordinates for Ti[(H$_2$O)$_6$]$^{3+}$ HS

Ti	-0.00000005237237	0.00000107917573	0.00000076744200
O	1.43389448126672	-0.95314262082181	1.16049709563268

O	0.10661021226397	1.71954926384705	1.16045836597408
O	1.54239486053507	0.76555109189948	-1.16099120612878
O	-1.54239520491229	-0.76555048381122	1.16099136765220
O	-0.10661117761060	-1.71954740833086	-1.16045688543705
O	-1.43389426518450	0.95314417595054	-1.16049710620011
H	1.73772142743009	-0.70705244943129	2.05676437047609
H	1.97117137009056	-1.72565954645467	0.89373800921245
H	0.50623922500634	2.57140405908830	0.89359750055056
H	-0.25702150259166	1.85898379633099	2.05738899747051
H	1.48043467099184	1.15425662417627	-2.05611581693214
H	2.48062910607568	0.84200906068928	-0.89556060040821
H	-1.48043459263930	-1.15426058980232	2.05611390439645
H	-2.48062782636036	-0.84201539736315	0.89555714022566
H	-0.50624054887341	-2.57140195901275	-0.89359585857491
H	0.25702235768577	-1.85898289485507	-2.05738671043210
H	-1.73772086994909	0.70705315343345	-2.05676431412341
H	-1.97117167085243	1.72566104529203	-0.89373902079595

19

Coordinates for $V[(H_2O)_6]^{2+}$ HS

V	0.02271288276455	0.01168859848532	-0.01866255179670
O	1.50902794762027	-0.91780637401477	1.20527097454864
O	0.10627177908616	1.74963124169839	1.22466255366350
O	1.56899580443413	0.80694830738995	-1.26308647827232
O	-1.52297334854952	-0.78369761970068	1.22546786159434
O	-0.05969497767923	-1.72595998946406	-1.26248698736814
O	-1.46360547925062	0.94117320502670	-1.24266604450966
H	1.91796638148410	-0.53446521189880	1.99550775737692
H	1.87279247037013	-1.80952745433781	1.10014723567714
H	0.68282038648306	2.51949615635128	1.10867961629116
H	-0.44851904016843	1.92319351408772	1.99973699033954
H	1.45489697282010	1.37680651449208	-2.03816570601192
H	2.52186033969499	0.68739981980880	-1.13488902417946
H	-1.40892888786078	-1.35219931030674	2.00155973399894
H	-2.47587755815232	-0.66536940815995	1.09635634962422
H	-0.63610870260901	-2.49585565252929	-1.14600312613857
H	0.49444108990975	-1.89948276011228	-2.03802908010844
H	-1.87177189355658	0.55840154235108	-2.03357678290876
H	-1.82796878484074	1.83257705183308	-1.13691678182043

19

Coordinates for $V[(H_2O)_6]^{3+}$ HS

V	0.00000044661959	0.00000030978906	0.00000022378908
O	1.42700699436572	-0.87950960635496	1.14950180280940
O	0.09652735564964	1.64427007687475	1.19116893428669
O	1.46683521631134	0.74197521599052	-1.19610339286566
O	-1.46683482588253	-0.74197453664032	1.19610306708007
O	-0.09652675948448	-1.64426930377035	-1.19116914283815
O	-1.42700672865961	0.87950907472303	-1.14950193540747
H	1.72485605583118	-0.58792090892444	2.03396325281854
H	1.91016448564231	-1.70445131913066	0.94403812341267
H	0.78562205003392	2.33767362295837	1.16747339874248
H	-0.55851022919089	1.91649000626313	1.86410823476028
H	1.34429631735189	1.17163003817018	-2.06588663824345
H	2.42257393405923	0.77791097155344	-0.99291571948127
H	-1.34429686332011	-1.17162853895607	2.06588685991264
H	-2.42257337825027	-0.77791020450911	0.99291448177162
H	-0.78562111448768	-2.33767317403611	-1.16747298299427
H	0.55851080837325	-1.91648949831100	-1.86410835700832
H	-1.72485789101127	0.58791792890101	-2.03396189018155
H	-1.91016587395123	1.70444984540954	-0.94403832036331

55

Coordinates for $Co[(H_2O)_{18}]^{2+}$ HS

Co	0.00000164735690	0.00000395517943	0.00000789497750
O	1.65054093926135	0.40948631876722	1.20280099560457
O	0.47170063345277	1.63731798891887	-1.19506317232386
O	1.17810830724703	-1.22021842153191	-1.20743011504666
O	-1.17810646751399	1.22022581047205	1.20744407053232
O	-0.47169914292775	-1.63731119178665	1.19507662778277

O	-1.65053651718324	-0.40947977794815	-1.20278706529981
H	1.96920316205685	1.31481148586295	1.39490843256829
H	2.36079669050631	-0.24133047171284	1.38048822445189
H	-0.15754317511537	2.36042124430534	-1.39297260668913
H	1.38828338929094	1.92324711055149	-1.38747380530684
H	0.97014268449734	-2.15892750649629	-1.39294128784028
H	2.12036173683706	-1.03842606950264	-1.40141866214811
H	-0.97014340086636	2.15893691503332	1.39294775058272
H	-2.12036006396765	1.03843088236596	1.40142812103993
H	-1.38828229580145	-1.92324700975772	1.38747635341879
H	0.15754217740999	-2.36042052947491	1.39297295411969
H	-1.96919630394518	-1.31480452057861	-1.39490005316121
H	-2.36079339618439	0.24133482905823	-1.38047834684456
O	2.34798290042583	3.09922399480423	1.19181680463677
O	3.47978968506774	-1.66571326841318	1.24298179568531
O	-1.49672463432089	3.58951961596403	-1.19366009626635
O	3.18841546317146	2.18271571866194	-1.24816664761014
O	0.32178554128960	-3.85998205920024	-1.25234176531533
O	3.85728265989415	-0.50841409178564	-1.19757216166477
O	-0.32178472899688	3.85998812196042	1.25233481971436
O	-3.85728233492857	0.50840678133448	1.19757604821239
O	-3.18841614983721	-2.18272389086645	1.24815880523241
O	1.49672019859809	-3.58952504305162	1.19365505382840
O	-2.34797866682510	-3.09921812393329	-1.19182725382116
O	-3.47979505502282	1.66570865355928	-1.24297993272326
H	2.99947005967892	3.53342234330491	1.76016623120519
H	2.78561145294465	2.95728099743794	0.31831733367380
H	4.13715943821345	-1.85227304399888	1.92741059280409
H	2.86857422728701	-2.44304987749902	1.22012623922015
H	-1.16439881884912	3.90374715014344	-0.31809157732176
H	-1.55333732341070	4.36711013977181	-1.76642138689200
H	3.55114851200879	1.26340432041517	-1.23433503579939
H	3.68095897189082	2.66372790500467	-1.92732503682298
H	-0.65670256291191	-3.72237420501504	-1.23167776290860
H	0.49289359136079	-4.52268981706488	-1.93560972038796
H	3.95378724442686	-0.95773211722400	-0.32310067913040
H	4.55658169457116	-0.85620193018632	-1.76838646317018
H	-0.49288999304936	4.52269814250310	1.93560135584346
H	0.65670295466529	3.72237890174100	1.23166847788416
H	-4.55658255855515	0.85619062859875	1.76839141870523
H	-3.95379035133080	0.95772520366396	0.32310543089747
H	-3.68095920182587	-2.66373829626074	1.92731600155313
H	-3.55114936003295	-1.26341255460372	1.23433141834911
H	1.55333117689199	-4.36711698776305	1.76641454020100
H	1.16439551688690	-3.90374924587266	0.31808478674630
H	-2.78560972825429	-2.95728285693113	-0.31832801614740
H	-2.99946326773937	-3.53341268561061	-1.76018249057669
H	-2.86858218806453	2.44304717979824	-1.22012738531800
H	-4.13716496972909	1.85226325488799	-1.92741005293438

55

Coordinates for Co[$(H_2O)_6$]$^{2+}$ LS

Co	-0.00000446001452	0.00000209818807	0.00000410257486
O	1.45853793939177	0.44783992801125	1.28909138487768
O	0.47869011258648	1.46306891368311	-1.26292906792335
O	1.33374446837319	-1.40158589926074	-0.98860982576342
O	-1.33374690475663	1.40158891265494	0.98862167128415
O	-0.47870244645276	-1.46306781604502	1.26293131016121
O	-1.45854095035830	-0.44783153662663	-1.28909210790857
H	1.82459570598272	1.35800177842928	1.34699298263179
H	2.19990160958798	-0.19766911558506	1.34247690908390
H	-0.13063895747783	2.23529336053914	-1.31587138088684
H	1.40564207115746	1.77828711573953	-1.34928252827711
H	0.98993526154772	-2.20477474160243	-1.42643645382453
H	2.11162675347923	-1.08670316665258	-1.48262328640550
H	-0.98993463687883	2.20477950417937	1.42644232868928
H	-2.11162324454881	1.08670366158204	1.48264160227477
H	-1.40565528806421	-1.77828786327751	1.34927434538991
H	0.13063142794825	-2.23528714265282	1.31586991644337
H	-1.82459409766516	-1.35799504325668	-1.34699480182531
H	-2.19990578349089	0.19767613306960	-1.34247849675991
O	2.39451051497801	3.04934234550143	1.18966263041846
O	3.38423977558971	-1.53014755809130	1.18285681849548
O	-1.37663301428237	3.49928813933191	-1.14081921549082

O	3.14437688067999	2.20183414241955	-1.26018901418041
O	0.23375546449470	-3.92603559550421	-1.31922630799618
O	3.93291628781523	-0.38775107194508	-1.25735568870062
O	-0.23374941286647	3.92603951679889	1.31922345653209
O	-3.93292062628350	0.38773542247332	1.25735974217929
O	-3.14437712811036	-2.20184831948832	1.26018144592462
O	1.37664229708054	-3.49928042846057	1.14082727086202
O	-2.39450539069970	-3.04933803720966	-1.18967713131753
O	-3.38424317158381	1.53014996782713	-1.18285177567837
H	3.06346218763684	3.39137547015677	1.79915121787890
H	2.83466539853685	2.94076096648722	0.31123647211974
H	4.00140867289025	-1.71695234442770	1.90410342069566
H	2.78110719452616	-2.30905532087273	1.12219077254823
H	-1.07442699756566	3.85075289567194	-0.26927704316102
H	-1.39568376652194	4.24952525845013	-1.75152581497930
H	3.57731990763007	1.30769933960676	-1.26337757410849
H	3.54810284518878	2.70799914562076	-1.97879982114316
H	-0.74177499985521	-3.78713920376483	-1.29630587633336
H	0.40089278557453	-4.62798365794118	-1.96270156486443
H	3.95460838780234	-0.84372526248641	-0.38256335315970
H	4.69665685765359	-0.70459121974444	-1.75871344059528
H	-0.40088306067988	4.62799276780254	1.96269396883295
H	0.74178102512472	3.78714149723044	1.29629835475725
H	-4.69666349279561	0.70457442328510	1.75871483949975
H	-3.95461055717008	0.84371285808786	0.38256987561495
H	-3.54810061290106	-2.70801596211616	1.97879184982220
H	-3.57732351221139	-1.30771489381906	1.26337471759343
H	1.39569349166235	-4.24951864889771	1.75153277407281
H	1.07443978778608	-3.85074753561045	0.26928583128903
H	-2.83466087683101	-2.94076480954160	-0.31125054529603
H	-3.06345588587657	-3.39136877326047	-1.79916839656844
H	-2.78111306342839	2.30905850863911	-1.12218420728859
H	-4.00141277333464	1.71695689667411	-1.90409729211159

55

Coordinates for $Co[(H_2O)_6]^{3+}$ LS

Co	-0.00000006694442	-0.00000210777677	-0.00000184055116
O	1.46908825105546	0.37310617462622	1.14766184960498
O	0.41878435263682	1.45301067212609	-1.15167438809614
O	1.05952478017235	-1.09674271011570	-1.13410107715550
O	-1.05952484278146	1.09673924971349	1.13409719523160
O	-0.41878390393133	-1.45301373334859	1.15167101396200
O	-1.46908659997380	-0.37311078935074	-1.14766683944041
H	1.83766834685181	1.29805734503299	1.22140531179605
H	2.19465446183447	-0.30600405714726	1.26440605027548
H	-0.19737136081067	2.23575837494835	-1.22178550483264
H	1.36886418469531	1.74543093437191	-1.26395065658267
H	0.83374802777811	-2.06345685650634	-1.25693949926913
H	2.04382531055581	-0.94985402455076	-1.21547631806474
H	-0.83374810406647	2.06345294169060	1.25693749695756
H	-2.04382568906547	0.94985242045943	1.21547293983959
H	-1.36886482477263	-1.74542922651571	1.26394970419167
H	0.19737203371519	-2.23576124702800	1.22178424576103
H	-1.83766938428298	-1.29806178062819	-1.22140688697386
H	-2.19465197410769	0.30600037008783	-1.26441078457643
O	2.34383557049530	2.89508396671593	1.25360292395171
O	3.33457439952306	-1.51634456350773	1.32446206288945
O	-1.32382120708153	3.47215373728485	-1.25078124919753
O	2.98654406057803	2.14122724478089	-1.32521400663011
O	0.35879982631598	-3.65646391900676	-1.33205969114836
O	3.67479281863777	-0.58359522828927	-1.25905792128912
O	-0.35880159323731	3.65645957788132	1.33206278227962
O	-3.67479214983203	0.58360085912050	1.25905797136539
O	-2.98654634965231	-2.14121789659818	1.32521750142429
O	1.32382237990261	-3.47215515201867	1.25078263500706
O	-2.34383991012785	-2.89508315817373	-1.25359874373333
O	-3.33456974049166	1.51634143401821	-1.32446709595542
H	2.94260584790182	3.20670712079864	1.95096237943286
H	2.83501978951448	2.96133556866667	0.40682259771075
H	3.96436369088971	-1.54849177188581	2.06165491785419
H	2.83535571463575	-2.36349846266916	1.34731020846411
H	-1.13891969648209	3.93299500215760	-0.40450759539775
H	-1.29556492398492	4.14497676004349	-1.94975283708559
H	3.47346355653910	1.28721160749266	-1.35063490370924

H	3.32666503982557	2.67320779821283	-2.06175116107999
H	-0.62438593930907	-3.65231576461069	-1.35478093029148
H	0.64924742127178	-4.21594556836242	-2.06937003865511
H	3.98088289198327	-0.97590840309052	-0.41364197343021
H	4.24098169677691	-0.94663876002903	-1.95881013577523
H	-0.64924996901164	4.21594011912928	2.06937363809776
H	0.62438433496675	3.65231177081426	1.35478460924232
H	-4.24097911454575	0.94664736008308	1.95881025086941
H	-3.98088273636472	0.97591211121613	0.41364134284955
H	-3.32666619117005	-2.67319630012093	2.06175675543102
H	-3.47346461828656	-1.28720147161574	1.35063771780270
H	1.29556602715462	-4.14497662832340	1.94975573318244
H	1.13891989988062	-3.93299814960082	0.40451025001177
H	-2.83502453628243	-2.96133146890651	-0.40681843465341
H	-2.94261071532336	-3.20670640588753	-1.95095772425528
H	-2.83535052044842	2.36349519481518	-1.34731318116651
H	-3.96435805371981	1.54848988937652	-2.06166066649003

55

Coordinates for Cr[$(H_2O)_{18}]^{2+}$ HS

Cr	0.00000005312509	-0.00000758693890	-0.00000217653280
O	-0.47057888260532	-1.25991248352848	1.54906005073606
O	-2.05114140682232	1.19935668251960	0.43675657772371
O	1.06660947439925	1.24623496024114	1.23339101656542
O	-1.06660634273380	-1.24625142258114	-1.23339508231532
O	2.05114390169450	-1.19936658706124	-0.43676187688947
O	0.47058022527073	1.25989757269669	-1.54906300183221
H	-1.40962375315646	-1.35295945448651	1.83450051465420
H	0.12647527860741	-1.37414130451592	2.32311140004353
H	-2.56208837790882	1.66522286866731	-0.24664292435095
H	-2.26018341683385	1.59415853515837	1.30302380603656
H	2.03975045484076	1.34007095504554	1.10949148257859
H	0.83369052695863	1.38519658121916	2.17855157098154
H	-2.03974722919243	-1.34008559624118	-1.10949452539550
H	-0.83369114407843	-1.38520035588326	-2.17855843016404
H	2.26018991517233	-1.59415831796752	-1.30303257998340
H	2.56208830529928	-1.66523951574027	0.24663498225071
H	1.40962496405028	1.35295816163716	-1.83449844314290
H	-0.12647531172738	1.37414091846063	-2.32311097693409
O	-3.13760262313716	-1.14109329248007	2.11542841461742
O	1.36931292550503	-1.24699440814625	3.58339767408322
O	-3.54196139713703	1.24389256565592	-1.96846579112163
O	-2.45621142497651	1.27759699643303	3.20222019777118
O	3.72408560336059	1.17024823316351	0.63058420244848
O	0.26518377182672	1.21822368928316	3.85667169029597
O	-3.72408047127101	-1.17026459728635	-0.63057521475083
O	-0.26518408707900	-1.21820685982544	-3.85667625980514
O	2.45621074650864	-1.27757484893220	-3.20223100178656
O	3.54195153451288	-1.24391135057024	1.96846565825789
O	3.13760764915806	1.14110039861140	-2.11541829624909
O	-1.36931767121713	1.24700832123940	-3.58339013392702
H	-3.59848896080644	-1.74045170752891	2.71928512684457
H	-3.07438965264340	-0.26869917041723	2.57768093971506
H	1.43909036063679	-1.94571868908232	4.24870252681929
H	2.22982064534132	-1.24704638590095	3.08310769457125
H	-3.79099313568717	0.38481447349776	-1.55383293408830
H	-4.36765529711140	1.67705933211975	-2.22489557117508
H	-1.54290480205198	1.26809882152917	3.57064900655176
H	-2.98608778724011	1.83325022799995	3.78976811414601
H	3.65132939348943	1.09384070028024	-0.35135470715006
H	4.35251788323253	1.88517952047859	0.80356722654077
H	0.68488045383538	0.33641775517835	4.01018374826763
H	0.60971250630114	1.81477718637692	4.53607784385194
H	-4.35251037438899	-1.88519911238016	-0.80355331043585
H	-3.65132309326350	-1.09385062392980	0.35136299090401
H	-0.60970867598080	-1.81475542296449	-4.53608888452329
H	-0.68488239763840	-0.33640074859822	-4.01018266324736
H	2.98608520622283	-1.83322244142412	-3.78978591209686
H	1.54290183637695	-1.26807580823189	-3.57065428850920
H	4.36764194887830	-1.67708279752318	2.22489897331543
H	3.79099010808792	-0.38483418844682	1.55383495961630
H	3.07439352129572	0.26871056237169	-2.57767938166772
H	3.59849412612458	1.74046416667833	-2.71926953250476
H	-2.22982671668198	1.24704933737080	-3.08310301719446

H	-1.43909888674224	1.94573555469947	-4.24869147241460

55

Coordinates for $Cr[(H_2O)_{18}]^{3+}$ HS

Cr	0.00000012298415	0.00000012166099	0.00000000362524
O	1.93462446601077	0.28111457794726	0.27842196964228
O	-0.29902181134835	1.94058476069818	0.20821878208169
O	0.26041243737425	0.23549156822322	-1.94303291463123
O	-0.26041142459978	-0.23549188909748	1.94303294913807
O	0.29902100943669	-1.94058545670844	-0.20821902178104
O	-1.93462546118100	-0.28111455165648	-0.27841946478173
H	2.30323784683234	0.98321642267650	0.88329741091675
H	2.62660799073241	-0.03304361361717	-0.37077031187510
H	-0.92292091127765	2.32761656716737	0.88345428999529
H	0.35596333373519	2.62576370498907	-0.10905751842143
H	-0.03698124477017	-0.44170890116129	-2.61531575978820
H	0.93870028181196	0.85400198604296	-2.33291707842092
H	0.03698167353448	0.44170822477181	2.61531594506792
H	-0.93869771544547	-0.85400382730320	2.33291800605835
H	-0.35596514382111	-2.62576421751825	0.10905541180200
H	0.92292065490509	-2.32761707743526	-0.88345407214578
H	-2.30324010619435	-0.98321331499425	-0.88329778746962
H	-2.62660791261345	0.03304221087708	0.37077398547469
O	2.63849922711070	2.35652671884699	1.79464523204260
O	3.53701423930443	-0.48089652044525	-1.69482842566889
O	-1.87387375556756	2.70384340506896	2.21691821077317
O	1.68341236349579	3.53142713617292	-0.55210055253326
O	-0.44735316640749	-1.80351704778308	-3.48637355453538
O	2.27762717681666	1.80245353415460	-2.70305138781333
O	0.44735280588088	1.80351817535619	3.48637340820517
O	-2.27762059253445	-1.80245689036906	2.70305247686334
O	-1.68341751148523	-3.53142641279929	0.55209471837972
O	1.87387477046594	-2.70384326063820	-2.21691732953488
O	-2.63850146693580	-2.35651950615719	-1.79465126428442
O	-3.53701298036497	0.48089258431606	1.69483701159559
H	3.51581325324017	2.48339747104296	2.18980041088379
H	2.53081726397182	3.05522950716974	1.11343174268931
H	4.48530080552579	-0.66134217518177	-1.59862443945274
H	3.13076742491614	-1.30546269894274	-2.04560692867868
H	-1.21150777733762	2.62573654228220	2.93724459065547
H	-2.27835867284604	3.58195539065655	2.30197955265720
H	2.05919793854484	3.10130338005919	-1.35333521633393
H	1.58349693378078	4.47220665230546	-0.76665157882823
H	-1.25760231522993	-2.17015848556447	-3.06537347276903
H	-0.63617820553874	-1.73919637448430	-4.43572455002072
H	2.99927796517557	1.14776754843338	-2.58344908998559
H	2.38477527989060	2.17834013538989	-3.59140194204817
H	0.63617944108315	1.73919709194661	4.43572404093342
H	1.25760041393897	2.17016180155957	3.06537260224759
H	-2.38476525875099	-2.17834601117161	3.59140243022740
H	-2.99927256498772	-1.14777143783973	2.58345392604534
H	-1.58350503382795	-4.47220691652289	0.76664287895123
H	-2.05920005439422	-3.10130397609036	1.35333160165475
H	2.27835919551499	-3.58195545524901	-2.30197923756607
H	1.21150841901857	-2.62573579546538	-2.93724348058911
H	-2.53082202005486	-3.05522443707330	-1.11343940736143
H	-3.51581511734360	-2.48338723607189	-2.18980827801108
H	-3.13076631376577	1.30545946127743	2.04561366980741
H	-4.48530019640887	0.66133680624820	1.59863680691523

55

Coordinates for $Cu[(H_2O)_{18}]^{2+}$

Cu	-0.00000024927026	0.00001612687818	0.00000930742404
O	-1.37169301205469	-1.31165394839702	0.58987052894960
O	-1.33704481721942	1.30079181980240	-0.69569395257971
O	-0.07773954743454	0.96987660843253	2.11853570655048
O	0.07773911001162	-0.96986476482918	-2.11850811189465
O	1.33704596998148	-1.30076242820271	0.69570553091757
O	1.37169773037005	1.31168353364679	-0.58985597470725
H	-2.25205543411481	-1.34383310684322	0.14920062227784
H	-1.49529280088416	-1.39202462312544	1.56609090670769
H	-1.38762786398134	1.39243516876269	-1.67800190562505
H	-2.24848141549913	1.32626072209723	-0.32210483391990

H	0.71306742209348	1.38229112724323	2.51285533614017
H	-0.85572046038191	1.47495383190432	2.40730559262598
H	-0.71306472339071	-1.38227347033636	-2.51284060858689
H	0.85572004088225	-1.47493441694426	-2.40729333544076
H	2.24847808259316	-1.32625315383323	0.32210771034236
H	1.38763092821635	-1.39242059540827	1.67801183068461
H	2.25206551322636	1.34383757860779	-0.14919471875415
H	1.49529473361783	1.39204448680911	-1.56607710622082
O	-3.77834140706206	-1.19587925408806	-0.73352781491432
O	-1.50065221162072	-1.33978129198638	3.32388242395692
O	-1.32027511279334	1.32480987489403	-3.43092847007453
O	-3.81068876796579	1.20086322845866	0.49313422303906
O	2.49455715386114	1.13481957915101	3.20795845863385
O	-2.79033756787905	1.07519630043420	2.98002243655908
O	-2.49453556616821	-1.13481866829152	-3.20795915428605
O	2.79030779537744	-1.07520966384538	-2.98003616106622
O	3.81067428727409	-1.20088429886959	-0.49315368364351
O	1.32027041042249	-1.32480023558574	3.43093463403125
O	3.77836411576787	1.19585793582249	0.73351166503142
O	1.50065123436505	1.33978544639100	-3.32387346993339
H	-4.48107822219921	-1.83941906885799	-0.56533919875103
H	-4.06557689298164	-0.34174336847758	-0.32694185588954
H	-1.79561890217212	-2.11208716132127	3.82646403026085
H	-0.53525171427096	-1.24190779876345	3.50208405573489
H	-1.78878439658159	0.46385819906121	-3.55973854919212
H	-1.79640075424852	1.98210060045972	-3.95761692665526
H	-3.56035405914802	1.16032238255535	1.45652478777268
H	-4.44151672044115	1.92821260090029	0.40039432719001
H	3.09360982025169	1.17402572269284	2.42772759190319
H	2.89729328332023	1.68007917288537	3.89727489516529
H	-2.44956007780692	0.19952349063190	3.27779452276131
H	-3.22274250498051	1.48053951888833	3.74402545629574
H	-2.89725739764813	-1.68008337149399	-3.89727970656652
H	-3.09359079785359	-1.17403450913796	-2.42773037454234
H	3.22269919833221	-1.48056568640100	-3.74404001455083
H	2.44954320370686	-0.19953149909998	-3.27780827785267
H	4.44149528311889	-1.92824037948159	-0.40041795051672
H	3.56033289556551	-1.16034253358452	-1.45654225948172
H	1.79639241584826	-1.98209393287510	3.95762298830810
H	1.78878695014507	-0.46385273031325	3.55974510238303
H	4.06558367599680	0.34171887040893	0.32692115816115
H	4.48111003162263	1.83938685139425	0.56531959525958
H	0.53525142513391	1.24191599356252	-3.50207415701493
H	1.79562068694979	2.11208918761765	-3.82645685240684

55

Coordinates for Cu[$(H_2O)_{18}]^{3+}$

Cu	0.00007499063120	-0.00002091223245	-0.00014255134431
O	-1.37885860386114	-1.31273104559664	0.55774375718417
O	-1.31308656120760	1.29895227807259	-0.72667050469270
O	-0.11630367987645	0.79286886313479	1.79301509716108
O	0.11650545660131	-0.79290854306484	-1.79333707616864
O	1.31323383215283	-1.29900155597556	0.72632180966828
O	1.37898642046189	1.31262725680298	-0.55821319228129
H	-2.27561571337736	-1.32243759539252	0.11217824738426
H	-1.51584767769292	-1.35484813828465	1.55180895840332
H	-1.31531508182333	1.36337505929987	-1.72660275517867
H	-2.26094133414327	1.30871245769065	-0.39970010387807
H	0.69626899970866	1.22382365486923	2.19358717701376
H	-0.94799089969864	1.26607213176946	2.06333158598796
H	-0.69600793466436	-1.22413820523060	-2.19371166366980
H	0.94825922545446	-1.26615752838458	-2.06336787919672
H	2.26113831679552	-1.30826260392978	0.39949449993789
H	1.31538722529311	-1.36345583477940	1.72627552296655
H	2.27544223641914	1.32339133673201	-0.11209264575885
H	1.51640242456522	1.35451204591073	-1.55225117605959
O	-3.71301027747362	-1.29605237072529	-0.72229877080902
O	-1.65098145884050	-1.30259178787774	3.21416205829180
O	-1.21878383940780	1.34229470066248	-3.40732535149287
O	-3.78508896703528	1.27600768386512	0.25759606133955
O	2.12585087127473	1.33168769454602	3.02391204940158
O	-2.54698587177325	1.33072394114785	2.73877067489945
O	-2.12582819879930	-1.33219882695155	-3.02379648434632
O	2.54736985957511	-1.33095461182008	-2.73871276021786

O	3.78540204540961	-1.27553377166442	-0.25758677074798
O	1.21854361571694	-1.34291031283935	3.40682125546560
O	3.71293348427467	1.29629601466723	0.72241787901520
O	1.65064537685434	1.30212664447620	-3.21443045440186
H	-4.37477329038276	-1.99095745870485	-0.57738905385186
H	-4.11842247088774	-0.44778873089813	-0.43688497409794
H	-2.09618928971657	-2.02726157868181	3.68119220869264
H	-0.71562332760271	-1.31437264062311	3.51552724837693
H	-1.65534757630627	0.49938307449690	-3.64950660552526
H	-1.65014911094950	2.03781225710347	-3.92907708031741
H	-3.60702037745553	1.34832691985915	1.22439687852044
H	-4.41483386155045	1.98070563983357	0.03785502023881
H	2.87680689470581	1.38573473231130	2.39143170321202
H	2.27343717605841	2.02207026020742	3.68897301647188
H	-2.49459836829413	0.47361208085795	3.21371135811209
H	-2.79495045022976	1.99952715928356	3.39702350603796
H	-2.27353531622122	-2.02269856506263	-3.68871004360916
H	-2.87684029661170	-1.38595612502271	-2.39136528692382
H	2.79532435646461	-1.99998552367011	-3.39673896801937
H	2.49527962429800	-0.47396406404642	-3.21388529903855
H	4.41527598916325	-1.98007849658555	-0.03773243870283
H	3.60749572444205	-1.34785532363382	-1.22441779792236
H	1.65034398377780	-2.03847612464777	3.92814887213710
H	1.65466397079550	-0.49988881472732	3.64940060607954
H	4.11816028875181	0.44808131174088	0.43653624747976
H	4.37482933987067	1.99114708028611	0.57785913260512
H	0.71516778316817	1.31262988290582	-3.51549698987936
H	2.09486232319833	2.02713492852035	-3.68186875395228

55

Coordinates for Fe[$(H_2O)_{18}]^{2+}$ HS

Fe	0.05767427053890	0.03088458500659	-0.02538418557089
O	2.12285853496763	0.41165424798885	0.19674686974165
O	-0.32686162730666	2.11528546092809	0.12955143192505
O	0.21795560838193	0.35298745120996	-2.12209819163665
O	-0.10245844684230	-0.29170595481879	2.07124379447474
O	0.44173820702738	-2.05369881459543	-0.18052055285459
O	-2.00746460072737	-0.35021194721280	-0.24749754393502
H	2.51079027955294	0.99084799838486	0.88254532887913
H	2.83081460432543	0.02217674602675	-0.35585446992113
H	-0.93703497942170	2.49324376048456	0.79596293720741
H	0.35393029058176	2.77872718584899	-0.10903817781342
H	-0.02531463764394	-0.34231848980856	-2.76818244436797
H	0.94326193054513	0.90024973465948	-2.49108408773330
H	0.14068710071249	0.40341298719508	2.71757644691529
H	-0.82775855765040	-0.83908496787643	2.44007982957345
H	-0.23880584856882	-2.71734603269255	0.05812301404810
H	1.05271912982742	-2.43179068782034	-0.84610979882129
H	-2.39538206349936	-0.92967062708311	-0.93309278443301
H	-2.71545823591761	0.03901784250914	0.30524645755698
O	2.66665194542207	2.55320234589743	1.86097791834768
O	3.65332988131114	-0.52868301537881	-1.89301857156249
O	-1.86000677884559	2.72845484768840	2.35230016974943
O	1.93369045313343	3.56670471483757	-0.56931675438144
O	-0.39597770980656	-1.95147453494587	-3.54525702878099
O	2.49822760842975	1.82949284904013	-2.68173315805721
O	0.51159718074253	2.01209019776990	3.49545271014212
O	-2.38275286370176	-1.76805092215055	2.63146842573859
O	-1.81833142880410	-3.50572833110385	0.51915362342109
O	1.97584045720086	-2.66738642155248	-2.40229356594292
O	-2.55125334425083	-2.49218146250633	-1.91098032187768
O	-3.53779210460263	0.59009406014638	1.84226161564493
H	3.51346767346155	2.83831024358599	2.23170813272731
H	2.51224699743152	3.10073678325790	1.05324931920167
H	4.58725693201900	-0.77744766625122	-1.92450319607706
H	3.13917082426075	-1.32107524698090	-2.18402891007319
H	-1.07434267439140	2.58208573276241	2.93399009684809
H	-2.21979424260601	3.59601666865467	2.58429632822304
H	2.23507477713001	3.01655398070361	-1.33280939837527
H	1.96102854196699	4.48698096340075	-0.86564392252087
H	-1.18890738856065	-2.25727062334298	-3.03826376666553
H	-0.63285979394549	-2.00038070941025	-4.48179713073765
H	3.08934125700234	1.05835618934967	-2.50663563825062
H	2.75145615622423	2.18074121687007	-3.54695567985542

H	0.74827223443578	2.06073551972736	4.43206060701387
H	1.30474378691784	2.31780005737479	2.98875898016176
H	-2.63591940406527	-2.11880402795046	3.49690792418616
H	-2.97376543151962	-0.99691951008316	2.45605751327081
H	-1.84564904091280	-4.42605710257049	0.81531745942534
H	-2.11973044619257	-2.95572229277838	1.28272454448633
H	2.33555696984868	-3.53495550578682	-2.63436600823950
H	1.19006632123415	-2.52098636221850	-2.98385711320771
H	-2.39725857452160	-3.03946495990005	-1.10298991970567
H	-3.39787976990354	-2.77732715933815	-2.28210744673747
H	-3.02386030294008	1.38262571273644	2.13327092175469
H	-4.47178375748500	0.83865809211145	1.87343226747124

55

Coordinates for $Fe[(H_2O)_{18}]^{3+}$ HS

Fe	-0.00026751185729	0.00050122443899	0.00005078617796
O	-0.49341258306491	-1.13333602273351	1.59104001835474
O	-1.62053222409764	1.14238200703390	0.37247431588150
O	1.13556088822085	1.13916244437515	1.21529365032426
O	-1.13606348488819	-1.13786351923122	-1.21525062464599
O	1.62000563162509	-1.14169377321668	-0.37182601598487
O	0.49343977211637	1.13415841557271	-1.59068800568936
H	-1.43485215489603	-1.35587575064760	1.83372404830032
H	0.13131917737050	-1.32363732694938	2.34867757744753
H	-2.30812347227018	1.35561274637304	-0.31807035046817
H	-1.96614561752642	1.32671106840286	1.29233854923941
H	2.10463333587647	1.32660968427922	1.05515918996668
H	0.87691020273745	1.35919849659069	2.15334422637110
H	-2.10470381780800	-1.32637562178250	-1.05382467223909
H	-0.87803573374378	-1.35810875460504	-2.15344607097740
H	1.96589411985130	-1.32635812246290	-1.29147306821845
H	2.30679454567104	-1.35598175223352	0.31922815675603
H	1.43510834921407	1.35615107308322	-1.83294025329638
H	-0.13072058857009	1.32410781873914	-2.34887255748077
O	-3.10039076956100	-1.32541311595009	2.12689416689811
O	1.30826019384307	-1.28796887769829	3.52633383084078
O	-3.38990648659944	1.32184144143167	-1.61114378808459
O	-2.41675517741877	1.28503032312661	2.90170159279071
O	3.72084226904198	1.29116142057770	0.63793163147035
O	0.29678777922414	1.32758799938402	3.73816229147725
O	-3.72137129168193	-1.29116354032473	-0.63845534575320
O	-0.29715654860456	-1.32759592777624	-3.73775665039465
O	2.41662437414335	-1.28579839685987	-2.90103737762854
O	3.39077992061210	-1.32185956701498	1.61002003242411
O	3.10048978954487	1.32456020555734	-2.12718268199559
O	-1.30726557307437	1.28814742028991	-3.52696473299122
H	-3.50719757324638	-2.00252081618903	2.69075515859143
H	-3.16881959709235	-0.47611065723000	2.61433164251851
H	1.33406271556172	-1.97551042915603	4.21015323241933
H	2.16919920215160	-1.33448139034066	3.05232044169473
H	-3.84842400189884	0.47316529705533	-1.42946909233470
H	-4.07950794603214	2.00136408820862	-1.68010075984389
H	-1.57741879566531	1.33201579512469	3.41238899200213
H	-2.99735356618536	1.97171794197063	3.26557046826856
H	3.74465493821035	1.33824797069015	-0.34448144899637
H	4.32792771487067	1.97541785156040	0.96062053532156
H	0.68421117922325	0.47857184042858	4.04200591031124
H	0.58329980259872	2.00546264559931	4.37090051593529
H	-4.32793342728661	-1.97559469630008	-0.96175725008726
H	-3.74598384131630	-1.33840563985532	0.34389884510706
H	-0.58401931878300	-2.00553895511222	-4.37025916167995
H	-0.68364618052829	-0.47836444642955	-4.04220405696075
H	2.99690405010434	-1.97294177037378	-3.26455400293601
H	1.57730337158144	-1.33267060934679	-3.41172589696433
H	4.08078760348854	-2.00100621329027	1.67843788721428
H	3.84854399286334	-0.47281098177895	1.42806746968636
H	3.16941839054669	0.47456476586625	-2.61334938579379
H	3.50742373941436	2.00101655670223	-2.69172445093790
H	-2.16790736614924	1.33491882870173	-3.05240622202547
H	-1.33327249986123	1.97559920372509	-4.21086123938264

55

Coordinates for $Mn[(H_2O)_{18}]^{2+}$ HS

Mn 0.00000841836590 0.00026698731554 -0.00057458697320
O 1.71535084516707 0.36904017809279 1.27943424258739
O 0.54796641246657 1.78939312287893 -1.10825084611772
O 1.21195802484235 -1.24327837208680 -1.31060100396278
O -1.21286303785878 1.24447702162366 1.30788619188372
O -0.54789479884260 -1.78878170666319 1.10746016311393
O -1.71616134624265 -0.36905177063270 -1.27971716087302
H 2.05073920407297 1.27147050531417 1.46033420054868
H 2.42033182714970 -0.28827307000305 1.45577411570364
H -0.08195041469536 2.44671909114796 -1.46350167636086
H 1.45305085262015 1.97208483010737 -1.43486712689363
H 0.98466200739345 -2.18467286277465 -1.46199423360285
H 2.17061384489074 -1.11068336908923 -1.46265861564813
H -0.98532521576365 2.18540153948564 1.46182979915132
H -2.17122101170668 1.11119368916889 1.46111040955833
H -1.45310719066121 -1.97150888089235 1.43376231312014
H 0.08194617974176 -2.44601350660406 1.46297775433078
H -2.05110671932279 -1.27155180534306 -1.46101073658856
H -2.42134260598103 0.28803093646225 -1.45602653076108
O 2.42222725755209 3.05059013728491 1.17557125297843
O 3.50501794491369 -1.73643602055307 1.25836585502981
O -1.50729353162613 3.63628962941796 -1.20606379039330
O 3.27263396051649 2.11330419938576 -1.27386629444111
O 0.26026800279729 -3.84419691643638 -1.22381948327072
O 3.90085270347109 -0.59017019571631 -1.18740599623000
O -0.25887807461735 3.84407845582146 1.22500341722281
O -3.90133287242852 0.58969747250429 1.18793469570350
O -3.27228152804126 -2.11354163602534 1.27340425771176
O 1.50705016120247 -3.63608023799624 1.20729553306590
O -2.42121157914597 -3.05102890104058 -1.17610641600829
O -3.50607717398511 1.73644021982799 -1.25789329136435
H 3.06710235121545 3.51829588437725 1.72451534139747
H 2.85349229335777 2.89717145839446 0.30148300644380
H 4.16844505015417 -1.95597360203231 1.92695218267494
H 2.87884852899651 -2.50232616246053 1.21982571407234
H -1.14489014065341 3.91099552745369 -0.32868477579615
H -1.59437510233037 4.44212821562021 -1.73418216593196
H 3.61315397968991 1.18723209615444 -1.22022360422761
H 3.81754448841609 2.56849850960429 -1.93040347456697
H -0.70654612708557 -3.65510635966450 -1.14709044351466
H 0.35245895823258 -4.53010280149523 -1.89936402158741
H 3.98131241165951 -1.03254114884869 -0.30744662628719
H 4.61380493683264 -0.93869691854483 -1.74070921787078
H -0.35014964017170 4.53075355435375 1.89988439108396
H 0.70774580728161 3.65420325313956 1.14783962013696
H -4.61397629680790 0.93801569664890 1.74176773117155
H -3.98243701990786 1.03221344777423 0.30813584433007
H -3.81675067439448 -2.56908204401838 1.93006476822862
H -3.61312532858899 -1.18756688455036 1.22012548763267
H 1.59388902868731 -4.44122255180160 1.73652148309120
H 1.14541588763155 -3.91193097109081 0.32994023472610
H -2.85277463770627 -2.89779883713617 -0.30216722552204
H -3.06572891547590 -3.51905312051636 -1.72520418161153
H -2.87961795001796 2.50202442681187 -1.21907846595753
H -4.16947243525936 1.95666056784453 -1.92628801433636

55
Coordinates for $Mn[(H_2O)_{18}]^{3+}$ HS

Mn -0.00000019775764 -0.00000019710367 -0.00000270568063
O 1.52358293497883 0.35314765476548 1.13633362935722
O 0.52321759855125 1.75455968176173 -1.15570420285956
O 1.09123504924029 -1.13662899312862 -1.12826789800008
O -1.09123597985475 1.13662858950474 1.12826126834450
O -0.52321321657936 -1.75455668666786 1.15570471414162
O -1.52358200046439 -0.35315122602453 -1.13633760750347
H 1.88471390648644 1.28751673971446 1.25264444804754
H 2.23448505642541 -0.34582145478430 1.29753567425255
H -0.10906420878124 2.45369229011198 -1.44088493992041
H 1.43565818442913 1.99978034854294 -1.43623993372687
H 0.85551639021401 -2.10885741053923 -1.27128121051260
H 2.06827421127418 -0.96380163919604 -1.28848669550489
H -0.85551798159199 2.10885673343024 1.27127716717569
H -2.06827546078337 0.96380217370453 1.28847944390130
H -1.43565310448812 -1.99977647533732 1.43624305510205

H	0.10906850594569	-2.45368841611216	1.44088739801701
H	-1.88471531098755	-1.28752024507678	-1.25264367183877
H	-2.23448396633472	0.34581714544794	-1.29754422924847
O	2.35177042100912	2.83972042344835	1.22104051600943
O	3.32051058313650	-1.53947126104504	1.31429137959790
O	-1.48399713083427	3.60117517068773	-1.35391843768849
O	3.19578336756427	2.26452540385910	-1.32331950995393
O	0.37369779068135	-3.64549063921431	-1.28463953021986
O	3.66422331331818	-0.59161432873101	-1.29962838742055
O	-0.37370374267932	3.64549025708684	1.28464597902809
O	-3.66422569018582	0.59161770742531	1.29961910151301
O	-3.19578285922302	-2.26452456164698	1.32332683326945
O	1.48400006501244	-3.60117531414422	1.35392184302918
O	-2.35177397303527	-2.83972287813205	-1.22102954829117
O	-3.32050513026721	1.53946872348140	-1.31430231714140
H	2.95570505066636	3.17712611853810	1.90155000234426
H	2.83715222066880	2.87543083343154	0.36185321825504
H	3.96894995176286	-1.56275480419154	2.03557051156920
H	2.81640390957339	-2.38984795352749	1.36420030506943
H	-1.26578847101507	4.00880493979952	-0.49203454160842
H	-1.57851693856782	4.32945225153028	-1.98825199910826
H	3.61455527241618	1.38098358883091	-1.37868011189530
H	3.67457625465752	2.83569714338403	-1.94400186237291
H	-0.60947174324010	-3.61180398374161	-1.26994427756781
H	0.61479377329258	-4.21253396793059	-2.03421135980992
H	3.95525432423691	-1.00286595147065	-0.45796678500213
H	4.23217801910719	-0.95561221141603	-1.99771221829729
H	-0.61480074551883	4.21253202343288	2.03421867079627
H	0.60946563149375	3.61180294131768	1.26995269255289
H	-4.23218243493460	0.95561768230058	1.99770012648432
H	-3.95525264155569	1.00286811103296	0.45795531796613
H	-3.67457411959340	-2.83569437311927	1.94401223329784
H	-3.61455702246451	-1.38098368576762	1.37868265345045
H	1.57852003405112	-4.32945269571428	1.98825501166371
H	1.26578710927350	-4.00880465532135	0.49203885171366
H	-2.83715442452316	-2.87542911051939	-0.36184098716301
H	-2.95571044071518	-3.17712953209982	-1.90153697842206
H	-2.81639830877372	2.38984542946888	-1.36420611880427
H	-3.96894168471714	1.56275454566362	-2.03558398038724

55

Coordinates for $Ni[(H_2O)_{18}]^{2+}$

Ni	0.00000330967117	0.00000049732650	0.00000438668642
O	1.81431202383900	0.45647185164345	0.90098460943965
O	0.33803156734721	1.54434909095612	-1.34672094560930
O	1.04050225726696	-1.19466836508354	-1.34935828555636
O	-1.04049864658899	1.19467093790179	1.34936279276797
O	-0.33802825848466	-1.54435000122737	1.34672524510406
O	-1.81430479729746	-0.45646940266314	-0.90098096161406
H	1.89874519208608	1.27874118547293	1.41963503502993
H	2.30341422679443	-0.26640048235506	1.34976852584314
H	-0.23865349807219	2.33831584040676	-1.37121284825947
H	1.27216941430013	1.84257705724136	-1.40973047879170
H	0.89204452637327	-2.16413320407217	-1.38987855492774
H	2.00605711881158	-1.02704036728617	-1.41703430392805
H	-0.89204283004220	2.16413615692007	1.38987977045792
H	-2.00605329244569	1.02704106854499	1.41703748442059
H	-1.27216718413415	-1.84257536805278	1.40973033065212
H	0.23865363051366	-2.33831915049678	1.37121423046248
H	-1.89874121412449	-1.27873867267435	-1.41963128482021
H	-2.30341176664405	0.26640087204317	-1.34976283682091
O	2.26956915453433	3.17661828228754	1.23322380077735
O	3.39978165610666	-1.72104928942986	1.31370516629488
O	-1.45108602857673	3.65350188867940	-1.17911045488704
O	3.04391904034347	2.17289268244935	-1.20062026603284
O	0.35429861098430	-3.88426979613009	-1.27057414610619
O	3.74523230501889	-0.55102690772638	-1.17395136082951
O	-0.35430326110802	3.88427372194525	1.27057177430000
O	-3.74522661878492	0.55102452923936	1.17395388360050
O	-3.04391723478701	-2.17288965901143	1.20061778826780
O	1.45108175931911	-3.65351062087527	1.17910717340323
O	-2.26957028783508	-3.17660934464743	-1.23322822899872
O	-3.39978751171261	1.72104129135165	-1.31370305262534
H	2.92804125060816	3.64586653278612	1.76400421402096

H	2.69149830339793	2.98721388188749	0.36011334157003
H	4.06669625561712	-1.88981486690455	1.99334286481613
H	2.82591435042751	-2.52397013933846	1.27493484900066
H	-1.14232272289165	3.97876375758800	-0.29777568762387
H	-1.44854999876183	4.41234200797230	-1.77911277940475
H	3.38784750783852	1.25235245092652	-1.11036158949761
H	3.55499457063177	2.58972048509611	-1.90817239815721
H	-0.63257707250921	-3.78470714213476	-1.25931322204893
H	0.56580330552533	-4.50657257713911	-1.98003296861720
H	3.86829408128986	-0.98358402564882	-0.29761656581038
H	4.44127769784080	-0.89147118927107	-1.75327781621650
H	-0.56580880929958	4.50657730323473	1.98002964079897
H	0.63257258623838	3.78471348346412	1.25930962126708
H	-4.44127201732646	0.89146832080533	1.75328064623718
H	-3.86829048882363	0.98357970721955	0.29761830723121
H	-3.55499423154062	-2.58971857423343	1.90816822478443
H	-3.38784345557237	-1.25234817643578	1.11036196874043
H	1.44854487370540	-4.41235297345525	1.77910663193616
H	1.14231802803333	-3.97876885592908	0.29777114829487
H	-2.69149973935121	-2.98720724430100	-0.36011720204866
H	-2.92804236831024	-3.64585517147054	-1.76401075778326
H	-2.82592138213949	2.52396313783019	-1.27493471898150
H	-4.06670388729983	1.88980354477355	-1.99333974020885

55

Coordinates for Ni[$(H_2O)_{18}]^{3+}$ HS

Ni	-0.00000043906136	0.00000025635011	-0.00000044709367
O	1.45989513906938	1.15489567373976	-0.64924437930455
O	1.31527948585372	-1.33715657640390	0.60898537074264
O	0.08784601265019	0.86189934890910	1.77339090309132
O	-0.08784583661024	-0.86189664992694	-1.77339305989734
O	-1.31528177177378	1.33715711406456	-0.60898395308612
O	-1.45989444835447	-1.15489598617376	0.64924423193968
H	2.30780530810287	0.78548660844110	-1.02353813024606
H	1.58052179052329	2.09340757462263	-0.32737496908087
H	1.31501502184405	-2.27430657540865	0.26467827917514
H	2.22365079339659	-1.07038544656395	0.93028349420557
H	-0.69733026255672	1.32681636582625	2.18207641548466
H	0.94516802816761	1.20585979822220	2.15115442277879
H	0.69733056494148	-1.32681422008296	-2.18207757389202
H	-0.94516791139056	-1.20585815471895	-2.15115570755355
H	-2.22365279560665	1.07038662298659	-0.93028428954748
H	-1.31501720873585	2.27430760321856	-0.26467871657635
H	-2.30780432650330	-0.78548706929579	1.02353836228150
H	-1.58052146305811	-2.09340775567523	0.32737449265759
O	3.68863656095597	-0.08207871409946	-1.44756249241906
O	1.66287425001657	3.56302638253894	0.46427613432709
O	1.15784660743634	-3.74602584070393	-0.52808147517576
O	3.70435480767071	-0.39946151625429	1.31633167412329
O	-2.11064907649395	2.12601900804921	2.57759863613758
O	2.46325264404266	1.84430243410747	2.49290242828606
O	2.11065044190715	-2.12601864437225	-2.57759873612938
O	-2.46325201176182	-1.84430361206067	-2.49290304734739
O	-3.70435434296131	0.39946034561612	-1.31633197723693
O	-1.15784812485995	3.74602764047424	0.52808014271327
O	-3.68863452512656	0.08207811754360	1.44756559539444
O	-1.66287602204261	-3.56302773439992	-0.46427683329164
H	4.37732015272345	0.33613145917357	-1.98871407125002
H	4.08012684405182	-0.23563204541686	-0.56033985225951
H	2.15651950561580	4.30103395969589	0.07316433597855
H	0.73102499259693	3.86645319067985	0.54995085854757
H	1.61502654004422	-3.53273882301851	-1.37051517379321
H	1.59264055429120	-4.53570256200770	-0.16821082124434
H	3.50727372361387	0.43325083983863	1.80230604866462
H	4.33255680180143	-0.89457289881849	1.86539629835704
H	-2.84217493626432	1.53079450464314	2.29570253071511
H	-2.29460855229296	2.36613082144410	3.49947480322028
H	2.42272365226180	2.67964759302171	1.97815785311456
H	2.69924099923085	2.08692244692604	3.40253425132319
H	2.29460993928650	-2.36613014028918	-3.49947499888019
H	2.84217690721503	-1.53079521033592	-2.29570208994520
H	-2.69923996422451	-2.08692462095051	-3.40253471053969
H	-2.42272244015771	-2.67964841389915	-1.97815768967205
H	-4.33255776731486	0.89457002747691	-1.86539652052037

H	-3.50727237759638	-0.43325203596859	-1.80230597272210
H	-1.59264229347399	4.53570456368369	0.16821030877429
H	-1.61502648935559	3.53274058695471	1.37051451289064
H	-4.08012637237207	0.23563148295185	0.56034375361186
H	-4.37731767772283	-0.33613108697963	1.98871849557476
H	-0.73102692826651	-3.86645500681353	-0.54995188582026
H	-2.15652170337252	-4.30103503056181	-0.07316505958598

55

Coordinates for Ni[(H_2O)$_{18}$]$^{2+}$ HS

Ni	-0.00000247016821	-0.00000236092020	0.00000088338111
O	1.66735232994358	1.14374310945574	-0.41440235193843
O	1.13694007468530	-1.34596489352248	0.67220222774442
O	0.00157229167980	0.70649334049338	1.75391257700313
O	-0.00157337077073	-0.70650297378574	-1.75390684431328
O	-1.13693346008799	1.34595635860099	-0.67220174366910
O	-1.66735918251948	-1.14374474903235	0.41441174373787
H	2.34953223195039	0.85873763198697	-1.06543192956707
H	1.65786428904251	2.13028077816526	-0.35432189585336
H	1.16531384466664	-2.24718596487834	0.22195985476179
H	2.08156187182595	-1.03754853677951	0.87486012623247
H	-0.79004884749284	1.24284058639511	2.07776493572965
H	0.86045713730813	1.15380211877998	2.03882811800087
H	0.79004120155506	-1.24284118582059	-2.07776355010491
H	-0.86045879915539	-1.15382111623567	-2.03881504117526
H	-2.08155083720861	1.03753447732037	-0.87486633535975
H	-1.16531076194390	2.24717779574593	-0.22195423601720
H	-2.34954834159183	-0.85872539562287	1.06543812113656
H	-1.65785470933012	-2.13027915172680	0.35435088602318
O	3.75875759504046	-0.12980625083359	-1.62147397006481
O	1.67833563318649	3.69319424055094	0.46612464186611
O	1.18256053405110	-3.70652138076375	-0.50073019328717
O	3.53927101523505	-0.44655952802947	1.21985723223809
O	-2.09101389994126	2.09068951967388	2.54386121090670
O	2.29502525108901	1.83575056414234	2.41928810911311
O	2.09097599167075	-2.09066219149532	-2.54386893926668
O	-2.29501005509177	-1.83577944122119	-2.41926221009492
O	-3.53925166241288	0.44655313359185	-1.21987695787228
O	-1.18256762916523	3.70653037027784	0.50075654426054
O	-3.75878347002041	0.12983066338924	1.62142171274423
O	-1.67830149745651	-3.69319738824219	-0.46610148609321
H	4.47371325856594	0.22927136817469	-2.17084791409251
H	4.14116380543048	-0.29001289735420	-0.73601988912164
H	2.20330664618566	4.45033179812869	0.16325109772417
H	0.75565718562913	4.00836903219178	0.55215523587236
H	1.67445353252642	-3.52441794281640	-1.32984887453845
H	1.63462989402825	-4.44476950120771	-0.06157342452589
H	3.33535800416510	0.42188327639895	1.63369009444259
H	4.10692529651814	-0.92248243925571	1.84643617674155
H	-2.86092934438785	1.50604963780001	2.32955137117942
H	-2.17566349831946	2.32082283259558	3.48283445820745
H	2.28744399315094	2.67858895260836	1.90935258090372
H	2.51038643793428	2.06121031633113	3.33826148228962
H	2.17559620976606	-2.32080609790504	-3.48285050642535
H	2.86089961840059	-1.50602138831985	-2.32957909524233
H	-2.51037657790247	-2.06125370571800	-3.33822374522334
H	-2.28742249341256	-2.67861033015190	-1.90932298829057
H	-4.10689837921577	0.92247079757863	-1.84646918926837
H	-3.33534382571236	-0.42189730006016	-1.63369327598665
H	-1.63462862572618	4.44478988349845	0.06159448411641
H	-1.67447684315352	3.52441424951540	1.32986777387387
H	-4.14116294033433	0.29002447232678	0.73597679608476
H	-4.47374924746694	-0.22923258663137	2.17075867459638
H	-0.75561610269883	-4.00834721104106	-0.55212091406014
H	-2.20325830254379	-4.45034739634678	-0.16324164945942

55

Coordinates for Ti[(H_2O)$_{18}$]$^{2+}$ HS

Ti	-0.00217063788754	-0.00011942999513	-0.00189872541647
O	1.71554806005395	0.41721380618239	1.24765933253834
O	0.49191008709004	1.72443390029925	-1.21299225182240
O	1.21842844533299	-1.25287561665299	-1.28177837555281
O	-1.22414405680693	1.25417771613738	1.27572503714714

O	-0.49763830576305	-1.72472540542074	1.20767529156659
O	-1.72218899417881	-0.41778630588753	-1.24910341910017
H	2.03781649289977	1.31994218684175	1.44708583618914
H	2.41809907203459	-0.23630428477578	1.44566882302625
H	-0.13372399754327	2.43881297670088	-1.44962967183293
H	1.40496454224190	1.98783647779257	-1.45015706215213
H	1.01580391958948	-2.19487986799087	-1.45719910384885
H	2.16729534387434	-1.08381634939564	-1.45674516854508
H	-1.02029434878922	2.19463642492709	1.45796189495060
H	-2.17125940130146	1.08252704505523	1.45690809133217
H	-1.41090645447763	-1.98859900193085	1.44386608662083
H	0.12939569661787	-2.43568267295502	1.45043892355519
H	-2.04281040913049	-1.32047533817914	-1.45037905819902
H	-2.42393952368722	0.23569327283328	-1.44940951484973
O	2.36423515594608	3.11213335186351	1.18083055290346
O	3.51255515312013	-1.67844112863177	1.25519633749856
O	-1.52398306945949	3.61683985157626	-1.18676818344704
O	3.21234867680170	2.18842693534616	-1.26111608033944
O	0.32132233859550	-3.87944984181951	-1.23953671729190
O	3.88406148655191	-0.50811339642270	-1.18857355320725
O	-0.31271439071763	3.87295324810847	1.25014920732487
O	-3.88888879497871	0.50753675328946	1.18407417809491
O	-3.21580584290298	-2.19063045026653	1.25559866908784
O	1.52793317844295	-3.60681974090146	1.19719469536627
O	-2.35607225361826	-3.11754827390790	-1.18415204304311
O	-3.50954854127748	1.68564393827533	-1.25657995109314
H	3.01145445643079	3.58500116838660	1.72252735115150
H	2.78900129295286	2.95962812583971	0.30245466193050
H	4.18087378528273	-1.88823027162553	1.92210858192336
H	2.89529174736566	-2.45104522976453	1.22404794091447
H	-1.17186181704126	3.90426156992004	-0.30896379041638
H	-1.61013236832240	4.41497284535822	-1.72657143489465
H	3.56250213447595	1.26457059712272	-1.22927421740255
H	3.73784112716227	2.65881843348545	-1.92283033858403
H	-0.65411511247666	-3.72139735951794	-1.20761089390015
H	0.46628876274422	-4.57284725921347	-1.89792755862170
H	3.96767287007703	-0.95096483912107	-0.30919131986520
H	4.60634087990017	-0.84560073531634	-1.73670154723159
H	-0.45421451387444	4.56312458011894	1.91264960990424
H	0.66236167287428	3.71360892913814	1.21352868696082
H	-4.61241933487678	0.84284018828854	1.73180207620068
H	-3.96880789036815	0.95425077557490	0.30637485391142
H	-3.74097297669248	-2.66288827338385	1.91627411298819
H	-3.56783921120122	-1.26758722605707	1.22511182537162
H	1.61584152421926	-4.40312664868327	1.73952370853231
H	1.17689983034728	-3.89902447321899	0.32035023871026
H	-2.78774479509468	-2.96600966103130	-0.30927334650162
H	-2.99831305707103	-3.59344691190413	-1.72924904310933
H	-2.89068438767179	2.45635283717332	-1.22098157169918
H	-4.18089324581464	1.90219805933545	-1.91819266473368

55

Coordinates for Ti[$(H_2O)_{18}]^{3+}$ HS

Ti	-0.00000305107716	0.00000179832554	0.00000134895314
O	1.62461382487498	0.39475371999242	1.18543841229834
O	0.49260819469463	1.65820918368590	-1.08127931848237
O	1.14739684684725	-1.21135112527852	-1.19632896645241
O	-1.14739755167824	1.21135383652854	1.19633474984402
O	-0.49261171823783	-1.65820773051088	1.08128130467283
O	-1.62461642867721	-0.39475240471729	-1.18543740799368
H	1.97864338163373	1.31327703679297	1.34996487700762
H	2.31293526625144	-0.29342195140421	1.41164745200400
H	-0.14537847428942	2.37920843980853	-1.33654271075136
H	1.42591558437060	1.90658775184992	-1.33622207147031
H	0.93430192827909	-2.17396013718074	-1.35975119063825
H	2.10153659569376	-1.02374560170885	-1.41531630640663
H	-0.93430072565965	2.17396262949298	1.35975621575334
H	-2.10153719173789	1.02374721588786	1.41532251002596
H	-1.42591879814877	-1.90658880718436	1.33622328440192
H	0.14537556931404	-2.37920649198569	1.33654422553143
H	-1.97864349924537	-1.31327644416958	-1.34996649279731
H	-2.31293731765796	0.29342377839102	-1.41164712446223
O	2.37899466943826	2.95345591297594	1.27320332854728
O	3.40184795422873	-1.56549971067364	1.32891259397805

O	-1.38646711531869	3.54051475034576	-1.29810979938408
O	3.08185931470342	2.18079632148018	-1.33333034373586
O	0.36727409397281	-3.75364414185032	-1.31376442622822
O	3.73296148759716	-0.57391406530925	-1.29912991576273
O	-0.36726959230502	3.75364513129757	1.31376650986304
O	-3.73296077224805	0.57391427403545	1.29913359889797
O	-3.08186188234734	-2.18079907608402	1.33332537966782
O	1.38646736647334	-3.54051320541521	1.29811273862379
O	-2.37899182356243	-2.95345632901727	-1.27321040826706
O	-3.40184852927246	1.56549860714573	-1.32891537941903
H	2.97683685884776	3.33750769325618	1.93417051249937
H	2.85589954433503	2.97772198729865	0.41551641770296
H	4.08329046338902	-1.66654726453661	2.01188846963724
H	2.87420834586392	-2.39654737034506	1.33762101578823
H	-1.17661439744456	3.96970573531924	-0.44074972310055
H	-1.42072676020909	4.24729880745075	-1.96236670743183
H	3.52385508365840	1.30186461011189	-1.34669388612369
H	3.50287196796897	2.71070907399630	-2.02807454452562
H	-0.61466344656570	-3.70254636219448	-1.31362445892812
H	0.60877889132419	-4.38014174979897	-2.01377719006309
H	3.96310902055363	-0.96978711229813	-0.43027240652540
H	4.38094615317975	-0.91052787017823	-1.93837495216853
H	-0.60877136972824	4.38014246800431	2.01378051494371
H	0.61466793013212	3.70254618217943	1.31362330068690
H	-4.38094595895892	0.91052566063259	1.93837936323062
H	-3.96311050594228	0.96978623345748	0.43027634999207
H	-3.50287605442878	-2.71071402428482	2.02806703192129
H	-3.52385650271313	-1.30186702236521	1.34669119565211
H	1.42072673887631	-4.24729691251402	1.96237009977469
H	1.17661621009987	-3.96970467066193	0.44075265592517
H	-2.85589829044686	-2.97772466029014	-0.41552450785982
H	-2.97683172875450	-3.33750831617718	-1.93417956687529
H	-2.87421052056355	2.39654711288890	-1.33762240786702
H	-4.08328927938311	1.66654460550258	-2.01189324410439

55

Coordinates for V[(H_2O)$_{18}$]$^{2+}$ HS

V	0.00040939404661	-0.00090153999836	0.00031517931924
O	-0.53031388809986	-1.27279724748800	1.61949725913878
O	-1.68658280646746	1.24053790878418	0.36034176474311
O	1.15697771376602	1.24596954295403	1.27917201689032
O	-1.15753053821221	-1.24026633135438	-1.28290222298433
O	1.68303097915408	-1.24873388408398	-0.36406577638299
O	0.52759888237817	1.26924167335369	-1.62144034710581
H	-1.45947797214412	-1.44328822599941	1.87560483892508
H	0.05817450018843	-1.42678172445000	2.38695577623349
H	-2.36672934228358	1.43008583389898	-0.31731992029249
H	-2.04236599393590	1.43032225658777	1.25243584000431
H	2.11284188643901	1.41295265303464	1.14847374506967
H	0.91017838429242	1.44393280537583	2.20519569941598
H	-2.10803997745872	-1.42928263181306	-1.14465847346104
H	-0.90889083466528	-1.44531280690291	-2.20673676942930
H	2.04798322606663	-1.41260355373047	-1.25784159378368
H	2.36435755305632	-1.43840224513820	0.31229295264636
H	1.45490416173982	1.45113736120541	-1.87553003647803
H	-0.06161375416626	1.42210898238074	-2.38865011971914
O	-3.25887365237532	-1.18911622734780	2.12030331139984
O	1.39570824644838	-1.23954352054503	3.60563327842781
O	-3.46621298714548	1.18965024066198	-1.76065075671442
O	-2.42198034842002	1.25121585042599	3.02531043980152
O	3.84104820724674	1.24158947411616	0.59592254697376
O	0.21096765854580	1.20571109719664	3.88283953559462
O	-3.83917014622072	-1.24865508594674	-0.59124819617886
O	-0.20732356342350	-1.20804942347049	-3.88487676185093
O	2.42589554697101	-1.23470120780716	-3.02815837880544
O	3.46315808212259	-1.20077896954678	1.75965374312879
O	3.25989004983428	1.20444889018056	-2.11668106903232
O	-1.40031785766051	1.23189566523123	-3.60575995022511
H	-3.75482239788511	-1.74742974589886	2.73534357685805
H	-3.14849072147940	-0.31190647130592	2.56084347419178
H	1.54152614981784	-1.91423089978187	4.28305999445167
H	2.21129595057704	-1.22138930210546	3.04683877631519
H	-3.79877043523462	0.31353457708273	-1.44747088331541
H	-4.24240801411540	1.75299590103282	-1.88729836277427

H	-1.53078289873829	1.22911041765921	3.45316528376499
H	-2.93573092636443	1.92192256116690	3.49602748368762
H	3.76228985948723	1.22478834507803	-0.38942363245228
H	4.50471495851312	1.91225439362534	0.80797590138677
H	0.64414514107697	0.32687563871352	4.00961505622514
H	0.49415814743374	1.76382121934624	4.62054217624278
H	-4.50659015042524	-1.91715890321591	-0.79841229225993
H	-3.76238638510619	-1.22311476654201	0.39418491673471
H	-0.48711719615946	-1.76584572944736	-4.62405170412898
H	-0.64381588775603	-0.33053090102708	-4.00996180106721
H	2.94182957040576	-1.90611490462410	-3.49539581454061
H	1.53323242015682	-1.22012117407470	-3.45345315114900
H	4.23728203559697	-1.76718050880606	1.88561989832758
H	3.79963294201881	-0.32545645447584	1.44929961233396
H	3.15064420822816	0.32967023047971	-2.56201744000092
H	3.75622992920658	1.76633458417220	-2.72811891784986
H	-2.21560470515629	1.21238825834977	-3.04623563785401
H	-1.54816220371594	1.90519822483362	-4.28410396839655

55

Coordinates for $V[(H_2O)_{18}]^{3+}$ HS

V	-0.00049127701621	0.00005424365475	0.00097876394919
O	1.61157617220874	0.38206617597796	1.12793077949906
O	0.47270886035618	1.57590875814902	-1.14443173413525
O	1.12832046555319	-1.20504020411602	-1.13633388155739
O	-1.12849789314040	1.20382518894321	1.13982104197371
O	-0.47397608998503	-1.57708379395483	1.14396459743949
O	-1.61234743443699	-0.38160318884578	-1.12640094286154
H	1.93175147987473	1.30149293956537	1.34205243623094
H	2.32104679975968	-0.29582618937235	1.32059870140579
H	-0.15795134612966	2.32249867482420	-1.34238043442903
H	1.41384416938173	1.84559924807821	-1.34273431843065
H	0.90043828445079	-2.15918457482964	-1.32849683562733
H	2.08547469990416	-1.02096732854442	-1.34605197814793
H	-0.89837061021816	2.15558886051212	1.33841297401330
H	-2.08657836992557	1.02110945442448	1.34664032549494
H	-1.41546209641700	-1.84742224927536	1.34051672424388
H	0.15761464983957	-2.32237607999669	1.34382524709789
H	-1.93145230971103	-1.30127132995525	-1.34179613847899
H	-2.32098627073995	0.29631869232547	-1.32146834238255
O	2.35129170977292	2.94168334770931	1.30164963251164
O	3.40325651518582	-1.56378150273097	1.30140169756640
O	-1.35635925670565	3.51035069053390	-1.29827067555128
O	3.05835472394082	2.17808241238498	-1.30560399931302
O	0.36711421151253	-3.74256692383124	-1.30896085680033
O	3.71622834118580	-0.57855606403101	-1.30956608844152
O	-0.36381204137323	3.74228294604823	1.31319012673280
O	-3.71732189192602	0.57909743500302	1.30800558280958
O	-3.05928099999663	-2.17716627847702	1.30151610813589
O	1.35501722113422	-3.51124028725302	1.30361562531736
O	-2.34901268961173	-2.94089267081834	-1.30599874144644
O	-3.40527716687936	1.56297584002184	-1.30277595010519
H	2.95249285360170	3.31109307737247	1.96787588807273
H	2.82906974559525	2.97064575339881	0.44453376075689
H	4.07531995553955	-1.64073078158948	1.99668250510319
H	2.86582376862908	-2.38725466873316	1.34229948769785
H	-1.14164949965819	3.94457963952475	-0.44425413218935
H	-1.37527282988122	4.21048061770001	-1.97012364263375
H	3.51050091582639	1.30504022007800	-1.33985384783405
H	3.46571115274600	2.72406953745814	-1.99619505466934
H	-0.61545161615780	-3.70178460128894	-1.34071967577184
H	0.63852126481098	-4.36062508801473	-2.00556014940148
H	3.98109794122471	-0.98500596631005	-0.45632729581417
H	4.33021216906777	-0.91592109737212	-1.98111912698642
H	-0.63214078198740	4.36622319333424	2.00562628572051
H	0.61884592007572	3.70138494897494	1.33969364714586
H	-4.33257346946011	0.91691507642420	1.97817871312579
H	-3.98149840913184	0.98414802242117	0.45385617474422
H	-3.46647211746249	-2.72402714911008	1.99152281626126
H	-3.51094316613308	-1.30389837718635	1.33758396505301
H	1.37370120008082	-4.20935530850016	1.97755705172957
H	1.14057714382726	-3.94757739074444	0.45069920110600
H	-2.82869432981823	-2.97315922842599	-0.45014799585602
H	-2.94898631121848	-3.30724903892484	-1.97503484655064

H	-2.86904670934417	2.38730776953073	-1.34037298887051
H	-4.07600535062051	1.64074459785876	-1.99924998665273
